# Weld Formation Between Polymer Films Prepared at Different Temperatures: Insights from Molecular Dynamics Simulations

**DOI:** 10.1021/acs.macromol.5c00569

**Published:** 2025-09-09

**Authors:** Mauro L. Mugnai, Jonathan E. Seppala, Peter D. Olmsted

**Affiliations:** † Institute for Soft Matter Synthesis and Metrology, 8368 Georgetown University, Washington, D.C. 20057, United States; ‡ Materials Science and Engineering Division, 10833 National Institute of Standards and Technology, Gaithersburg, Maryland 20899, United States; § Department of Physics, Georgetown University, Washington, D.C. 20057, United States

## Abstract

Inspired by Fused Filament Fabrication (FFF) Additive Manufacturing (AM), we use Molecular Dynamics (MD) simulations to investigate the early stages of the formation of the weld between two polymer films prepared at different temperatures – one above and one below the dilatometric glass transition temperature. We identify three stages of welding: (i) surface approach and formation of the initial contact, (ii) surface adjustment, and (iii) interdiffusion. Surface interactions affect film roughness, polymer conformation, and interfacial temperature during the initial stage. As the two layers come into contact, heat transfer equilibrates the system in an asymmetric way: the hot film cools down more slowly than the cold film heats up. When the films are allowed to exchange heat with the environment, most of the effects of the temperature difference at the interface terminate during the initial surface adjustment, before polymer interdiffusion begins at around the bulk Rouse time. However, if the films are isolated, the onset of interdiffusion occurs earlier for films prepared at different temperatures compared to films prepared at the same temperature. This indicates the importance of thermal relaxation across the interface between welding films, and suggests mechanisms to improve the weld strength.

## Graphical abstract

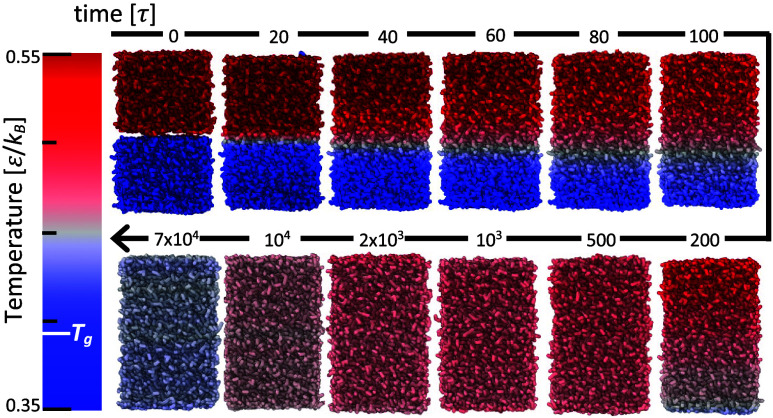

## 1 Introduction

In Fused Filament Fabrication (FFF) Additive Manufacturing (AM), three-dimensional (3D) structures are printed by depositing polymeric material layer-by-layer according to a predefined schedule. Focusing on amorphous systems, the dense polymeric fluid is extruded from the nozzle at a temperature *T*_h_ higher than the glass transition temperature (*T*_g_). A few seconds after deposition, the melt cools down to the ambient temperature *T*_a_ set by the machine configuration, vitrifies (*T*_a_ < *T*_g_), and forms the new layer (referred to as *p*th layer, or *L*_p_) of the designed structure. Next, a new layer (*L*_p+1_) is extruded and deposited on top of *L*_p_. Upon contact between *L*_p_ and *L*_p+1_, there is a temperature difference that can be ≈100 K or more^1^ over molecular distances. Before *L*_p+1_ vitrifies, it transiently raises the temperature of *L*_p_ above *T*_g_.^1^ This enables weld formation via interdiffusion between the two polymer melt layers,^1,2^ which leads to strengthening of the bonding between two layers via the formation of interfacial polymer entanglements.^3^ Notably, fewer entanglements are formed at the weld as compared to bulk,^4^ which correlates with the observation that the weld is often the failure point of the structure.^1,2^ Therefore, (i) tuning weld strength by adjusting processing conditions such as extruder temperature and velocity^5^ and (ii) understanding how interdiffusion across the interface occurs are important steps toward the design of 3D-printed objects.

Here, we address the following question: does the enormous temperature difference across the interface between a polymer melt and a glassy polymer affect the structure and dynamics of the polymers at the interface between the two layers? Despite being at the core of FFF, this question has remained largely unanswered. Theoretical models of welding progressively added information about thermal history and thermal gradients. Wool and O’Connor^6^ present a model for crack healing featuring a sequence of stages, including approach between the interfaces, wetting of the interfaces, and diffusion across the interfaces. They argue that temperature can impact all the steps of the healing process, for instance by slowing down wetting, diffusion initialization, recovery, and polymer conformation. Though they mention fast thermal quenching, they do not discuss thermal gradients. Prager and Tirrell^7^ and Kim and Wool^8^ developed models for healing polymer–polymer junctions based on polymer reptation^9^ across the interface in isothermal conditions. Yang and Pitchumani^10^ used a similar framework and considered a temperature-dependent diffusion coefficient. Following a similar idea, Seppala et al.^2^ introduced the equivalent isothermal weld time, which enables one to relate time-dependent interlayer temperature to welding time. Mcllroy and Olmsted^4^ accounted for both time and space dependence of the temperature, numerically solving the heat equation to describe the spatiotemporal evolution of the temperature field in *L*_p_ and *L*_p+1_, while simultaneously accounting for temperature (and thus time) dependence of the polymer relaxation times. This model could not describe the short-term, local dynamics for which microscopic descriptions provided by simulations are necessary.

Simulations of polymer interdiffusion have a long history. Binder and co-workers^11,12^ deployed lattice simulations to investigate the validity of theoretical models for asymmetric interdiffusion during welding of two films made of different polymers. More recently, Robbins and co-workers^3,13,14^ investigated the strength of a newly formed weld as a function of the length of polymer chains, the alignment of the macromolecules at the interface due to shearing of the melt during extrusion, and the time allotted for weld formation before a sudden (spatially uniform) quench to a temperature *T*_a_ < *T*_g_. Pierce et al^15^ investigated interdiffusion between two different polymer films, one fluid and the other glassy, at a given temperature, thus probing interdiffusion between different materials. Notably, all of these studies were conducted in (homogeneous) thermal equilibrium, in a few cases followed by a spatially uniform temperature quench. The role of the temperature difference across the interface between *L*_p_ and *L*_p+1_ was not explored.

Here, we perform simulations of the early stages of welding between two thin films made of short polymers and prepared at different temperatures, in contact with a low-density fluid designed to apply atmospheric pressure normal to the surface of the film, and to absorb the energy dissipated from the welding films. The cold (bottom, *L*_p_) layer is at a temperature *T*_c_ < *T*_g_, where *T*_g_ is the dilatometric glass transition temperature of the polymer model; the hot (top, *L*_p+1_) layer is at *T*_h_ > *T*_g_; the difference *T*_h_ – *T*_c_ is commensurate with experiments.^1^ Similar to the healing stages discussed by Wool and O’Connor,^6^ we observe that the early stages of welding feature: (I) surface approach and the formation of the initial contact, (II) surface adjustment, and (III) the beginning of interdiffusion. Initial contact occurs rapidly, and the thermal gradient enhances the asymmetry of polymer conformation and surface structure at the interface between the two juxtaposed layers. In addition, surface potential energy is released as kinetic energy upon contact, leading to a localized heating of the two adjoining surfaces. During surface adjustment, the contact area grows, and polymers move to attain bulk-like conformation before starting interpenetration. The dissipation of the thermal gradient is faster in the cold layer, and it is completed by the beginning of interdiffusion, which we identify as occurring at the bulk Rouse time. However, this observation is affected by the mechanism of energy dissipation into the environment: welding isolated films shows that system size and the presence of temperature difference at the interface affect the temperature of the films and the timing for the onset of interdiffusion. This highlights the importance of thermal transfer across the interface and allows us to predict ways in which stronger welds can be produced.

## 2 Simulation Method

Simulations are performed within the Large-scale Atomic/Molecular Massively Parallel Simulator (LAMMPS),^16^ using a variation of the Hsu-Kremer model^17–19^ (see Figure S1). This is a coarse-grained model that extends the Kremer-Grest^20^ model to allow more realistic internal pressures, simulation in contact with a free surface, and a more realistic glass transition temperature. Two free-standing films are prepared at different temperatures and then juxtaposed and simulated at constant energy in order to study heat diffusion. All quantities are provided in reduced units, in particular ϵ for energy, ϵ/*k*_B_ for temperature, σ for distance and hence ϵ/σ^3^ for pressure, *m* for mass and thus *m*/σ^3^ for density, and τ for time (see Section [Sec app1-sec1] for details). Full details and methods are described in the Appendix.

### 2.1 Single Film

We use *N* = 1000 polymers of *n* = 10 monomers for each film. The environment is represented by a fluid made of 907 particles (Figure 1a); the repulsive polymer-fluid interaction prevents fluid absorption into or adsorption onto the polymer film. The fluid (i) enables control of the pressure normal to the surface of the melt, and (ii) constitutes a tool to couple the system with a thermostat during welding. The films are prepared at high temperature and cooled down below the glass transition temperature in a stepwise fashion: the temperature is instantaneously decreased by 0.01ϵ/*k*_B_, after which the fluid and melt relax for 10^4^τ before a new temperature jump occurs, for an overall quenching rate of Γ = 10^–6^ϵ/(*k*_B_τ) (see Figure 1a). At every step, the temperature of the thermostat and the volume of the system are held constant, so the melt plus fluid are simulated in the *NVT* (constant number of particles, *N*, volume, *V*, and thermostat temperature, *T*) ensemble using the Nosé-Hoover algorithm. The volume is *V* = *A*(*L*_z,melt_ + *L*_z,fluid_), where *A* is the area of the film and is kept constant throughout this study, while *L*_z,melt_ and *L*_z,fluid_ are the thickness of the melt and of the height of volume occupied by the fluid. Both of these quantities depend on the temperature. The height of the fluid volume is selected using the equation of state of the fluid under the condition that at all temperature the pressure of the fluid ought to be equal to 0.01ϵ/σ^3^ (see Figure S2a), which is equivalent to about 1 atm (using the conversion in Table 1). As the system cools the polymer film becomes thinner so that *L*_z,melt_ decreases. This shrinkage is a result of our protocol; the fluid effectively acts as a barostat for the polymer film, which thus experiences roughly constant pressure, rather than constant volume, conditions (although we have not systematically tested whether the fluid-melt interaction is equivalent to an *NPT* ensemble for the film). The quenching is repeated 18 times starting from different initial conditions to generate 18 sets of independent high-temperature and low-temperature configurations to juxtapose in order to model weld formation (see next). The size of the box is slightly different in each repetition because the thickness of the melt can vary due to surface capillary waves.

**Figure 1:**
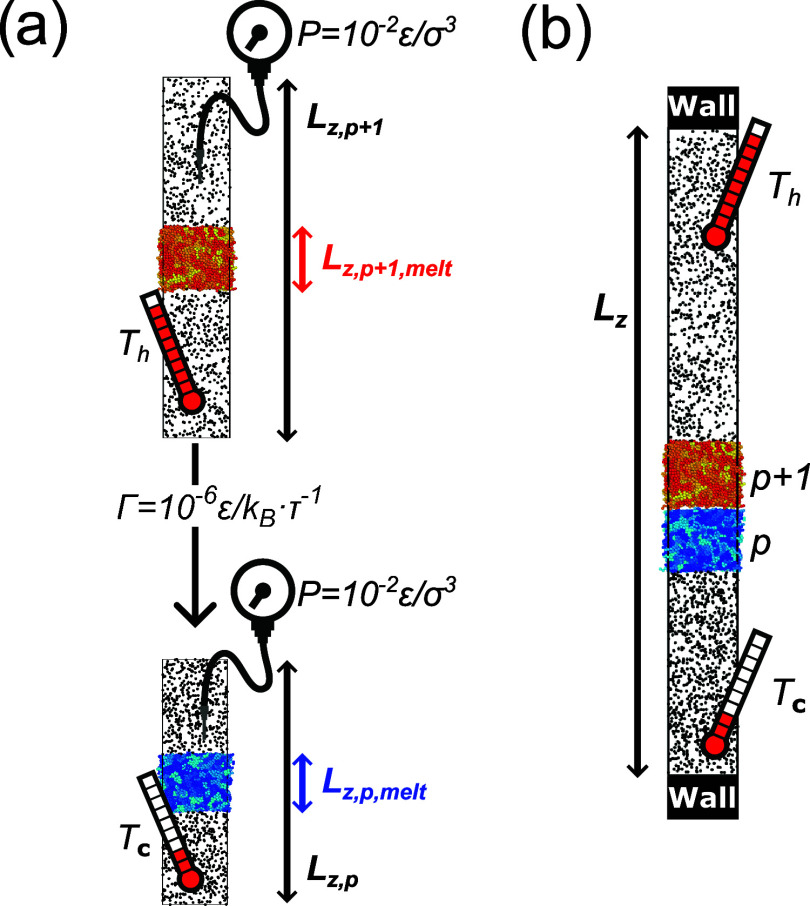
Model of single and double layer. (a) A polymer film at high temperature (top, red) is surrounded by a fluid (black) whose pressure is regulated by controlling its size *L*_*z*_. Periodic boundary conditions are applied. The system is cooled at fixed cooling rate to produce a new film (bottom, blue) and fluid at the same pressure normal to the surface of the film. (b) We juxtapose hot (*T*_h_) and cold (*T*_c_) films, allowing for a small gap between them, and enclose the system between rigid walls to avoid spurious heat exchange via the fluids, which are held at *T*_c_ for *z* < 0 and *T*_*h*_ for *z* > 0.

**Table 1:** Characteristic Scales and Conversion Units for the Coarse-Grained Model, Applied to the Reference Polymer Polystyrene (PS)^a^

name	experimental value
mass of Kuhn step (*m*_K_^exp^)	723.34 g/mol^†^
Kuhn length (*b*_K_^exp^)	1.780 nm^†^
bulk glass transition temperature (*T*_g_^exp^)	≈347 K*
bulk mass density (ρ_b_^exp^)	0.969 g/cm^3†^
thermal conductivity (κ)	0.128 J/(s K m)^‡^
thermal diffusivity (*D*_T_ = κ/(*c*_P_ρ))	0.076 mm^2^/s^□^

internal units	conversion
mass of a bead (m)	439 g/mol
potential well depth (ϵ)	11.3 pN nm
diameter of a bead (σ)	1.24 nm
time unit ( $\sqrt{m\sigma^{2}/\epsilon }$ )	9.99 ps
temperature unit (ϵ/*k*_B_)	816 K
pressure (ϵ/σ^3^)	5.89 MPa
density (*m*/σ^3^)	0.381 g/cm^3^

internal values	conversion
pressure = 0.01ϵ/σ^3^	0.0589 MPa
high temperature = 0.60ϵ/*k*_B_	490 K
low temperature = 0.20ϵ/*k*_B_	163 K
high *T* bulk density = 0.940 m/σ^3^	0.358 g/cm^3^
low *T* bulk density = 1.02 m/σ^3^	0.388 g/cm^3^
number of monomer in a polymer n = 10	
Kuhn lengths per polymer *N*_*K*_ ≈ 6.05	
bulk radius of gyration (*T* = 0.6ϵ/*k*_B_) = 1.45σ	1.798 nm
bulk Rouse time ≈ 7.3 × 10^4^τ (at *T* = 0.45ϵ/*k*_B_ = 367.2 K)	≈0.73 μs
thermal conductivity ≈ 5.52 *k*_B_/(τσ)	≈0.006 16 J/(s K m)
thermal diffusivity ≈ 1.34 σ^2^/τ	≈0.207 mm^2^/s

a The top part of the table contains experimental values (the superscript “^†^” refers to Everaers et al.,^21^ ^‡^ points to values taken from ref 22, taking data at 100 °C, and ^□^ to ref 23. at *T* = 108 °C and for molecular weight *M*_*w*_ = 2200 g/mol, whereas “*” indicates data extracted from Figure 2b of Baker et al.^24^ for a polymer of total mass M = *mn* ≈ 4390 g/mol) using WebPlotDigitizer.^25^ For the thermal diffusivity, note that if *κ* and *c*_P_ (the specific heat at constant pressure) were taken from ref 22, whereas ρ is from ref 21, the result would be *D*_T_ = 0.0719 mm/^2^s, fairly close to the value in the Table. The conversion strategy followed Everaers et al.^21^ We first determined the number of Kuhn steps (*N*_K_) and the Kuhn length (*b*_k_) using the following relationships:^26^ *b*_K_ = *R*_*ee*_^2^/*R*_*max*_ = 1.43 σ and *N*_K_ = *R*_*max*_^2^/*R*_*ee*_^2^ = 6.05, where *R*_*ee*_^2^ ≈ 12.44*σ* was obtained from simulations in bulk at *T* = 0.6ϵ/*k*_B_, and *R*_max_ = *b*(*n*–1), where *b* ≈ 0.964 σ is the average distance between two beads computed as the average distance between the first and the second beads of all polymers in bulk at *T* = 0.6ϵ/*k*_B_. To ensure that the Kuhn length of the simulated polymer matches the experimental value, we set the unit of length *σ* = *b*_*K*_^exp^ *N*_K_/(*b*_K_^*^(*n*–1)), where *b*_K_^*^ = *b*_K_/ σ. To ensure that the total mass is fixed, we set the mass unit (mass of a bead) *m* = *m*_K_^exp^ *N*_K_/*n*. To match the glass transition temperature in bulk we set ϵ = *k*_B_ *T*_g_^exp^/*T*_g_^*^, where *T*_g_^*^ = *k*_B_ *T*_g_/ϵ and *T*_g_ is in Table 2. The other quantities are derived.

For a subset of temperatures *T* > *T*_g_ we run longer simulations to extract the end-to-end distance autocorrelation function as a function of temperature. We perform these simulations in the *NVE* (constant number of particles, *N*, volume, *V*, and total energy, *E*) ensemble, where the volume depends on temperature as discussed before, and repeat the simulations 5 times to gather statistics.

We also prepare a larger system by copying the pre-equilibrated film 11 times in the *z*-direction. This thick film has 11,000 total polymers. In this case, we did not include the surrounding fluid, so the total pressure is 0ϵ/σ^3^. We equilibrate the thick film using both *NVT* and *NVE* simulations. The quenching of the thick film is performed using the Langevin algorithm in LAMMPS^16^ to control the temperature at each step. Given the large size of the system, we use a faster quenching rate of Γ = 10^–5^ ϵ/(*k*_B_τ).

### 2.2 Bulk

We compare the film to a bulk polymer melt made of 1000 polymers of length 10, which have a Kuhn length of *b*_*K*_ ≈ 6.05σ and a radius of gyration *R*_*g*_ ≈ 1.45σ at *T* = 0.6ϵ/*k*_B_. The bulk is cooled at the same rate as the thin film (i.e., Γ = 10^–6^ ϵ/(*k*_B_τ)), however it is coupled to a barostat via a Nosé-Hoover *NPT* (constant number of particles, *N*, external pressure, *P*, and thermostat temperature, *T*) algorithm, where *P* = 0.01ϵ/σ^3^ as for the film. Note that the pressure is isotropic in this case. We select a subset of systems prepared at temperatures *T* > *T*_g_ (*T* = 0.6ϵ/*k*_B_, = 0.55ϵ/*k*_B_, = 0.5ϵ/*k*_B_, and = 0.45ϵ/*k*_B_) and run longer simulations in the *NVE* ensemble to extract equilibrium properties of the melt. Again, the volume depends on the temperature. We did not repeat the calculation to gather statistics because the system is homogeneous and we gather statistics by averaging over the polymers. At the same temperatures, we conduct further simulations in bulk in the *NPT* ensemble in order to extract the thermal conductivity and thermal diffusivity.

### 2.3 Two Films

After quenching, we select a conformation of the film at high temperature, *T*_h_ = 0.55ϵ/*k*_B_ and a conformation at low temperature, *T*_c_ = 0.35ϵ/*k*_B_ (below *T*_g_, the glass transition temperature, see later). We place the two surfaces in near contact with each other (as close as possible while avoiding steric clashes, see details in Appendix [Sec app1-sec7]). We then run simulations using an energy-conserving algorithm (Velocity Verlet, as implemented in LAMMPS^16^) for the two films in order to avoid biasing thermal transfer, and we enclose the fluid between walls and couple it to a thermostat (via temperature rescaling) which keeps the temperature of the contacting bath constant (Figure 1b). We repeat this protocol for 18 different hot film/cold film pairs and we average the results over this ensemble. Following an identical procedure, we also prepare systems in which the two juxtaposed films are at the same initial temperature, and in this case we repeated the simulations 12 times starting two films at *T* = 0.45ϵ/*k*_B_. All of the simulations were repeated without the surrounding fluid or without coupling the fluid to a thermostat, thus performing the calculation in the *NVE* ensemble. We also constructed pairs of films at the same temperatures *T* = 0.50ϵ/*k*_B_, 0.55ϵ/*k*_B_, and 0.60ϵ/*k*_B_; in these cases we repeated the simulations 5 times. For the thick film, we only perform simulations in the absence of the surrounding fluid, and the protocol was repeated 5 times to gather statistics.

## 3 Results

### 3.1 Glassy Properties of the Thin Film

In order to characterize the glass transition temperature of the model polymer film, we monitor the density of the film as a function of the coordinate *z* perpendicular to the interface of the film, which we fit to the function^100^

1 $$\rho \left(z\right)=\frac{1}{2}\rho_{\mathrm{bulk}}\left\{\mathrm{erf}\left[\left(x+\mathrm{z̅}\right)/\delta \right]-\mathrm{erf}\left[\left(x-\mathrm{z̅}\right)/\delta \right]\right\}$$

where ρ_bulk_(*T*) is the density in the bulk (that is, in the middle) of the film, *L*_*z*,melt_(*T*) = 2*z̅* is the thickness of the film, and δ­(*T*) is related to the sharpness of the interface. In Figure 2a, we show that films become denser and thinner, and the interface sharper as the temperature is reduced, in agreement with intuition and with the literature.^27^ The density in the middle of the film is not far from the bulk density (Figure 2a), suggesting that the film is thick enough to have bulk-like properties in the middle. This is consistent with the fact that the radii of gyration of the polymers (*R*_g_ ≈ 1.45σ at *T* = 0.6ϵ/*k*_B_) are much smaller than the film thickness (≈ 20σ, see Figure 2a). Also, polymers at the surface have slightly smaller radius of gyration compared to those in bulk (see Figure 2a), which is consistent with the observation that the overall size of the polymers in a film decreases with film thickness.^19^ The fit of the data to eq 1 allows us to extract the bulk density of the film as a function of temperature. In Figure 2b we plot the logarithm of the bulk density as a function of temperature. Consistent with numerous other studies,^19,27–29^ we find a linear trend for high temperatures (liquid branch), a linear trend for low temperatures (glass branch) and a kink in between. The slope of the melt and glass branches is proportional to the thermal expansion coefficient α, with a ratio α_melt_/α_glass_, in line with the empirical rule that this ratio is ≈ 3 for most glasses in bulk^30^ (see Table 2). Although it is well-known that for films the ratio of α_melt_/α_glass_ depends on film thickness,^31^ in our case the protocols for thick and thin films differ owing to the different rates at which the films are quenched. The periodicity of the box in the *xy*-plane is tantamount to fixing the area of the film (a common procedure when studying polymer films^19,27,29^ ), which might quantitatively affect the relationship between density and temperature. To test this observation, we compare the polymer film with the melt in bulk (Figure 2b). The slopes of the liquid branches are nearly identical, whereas the thermal expansion coefficient of the glass is larger in bulk than for the film, leading to a slightly smaller α_melt,bulk_/α_glass,bulk_ (see Table 2 and the inset in Figure 2b), although still close to the empirical value of 3.^30^

**Figure 2:**
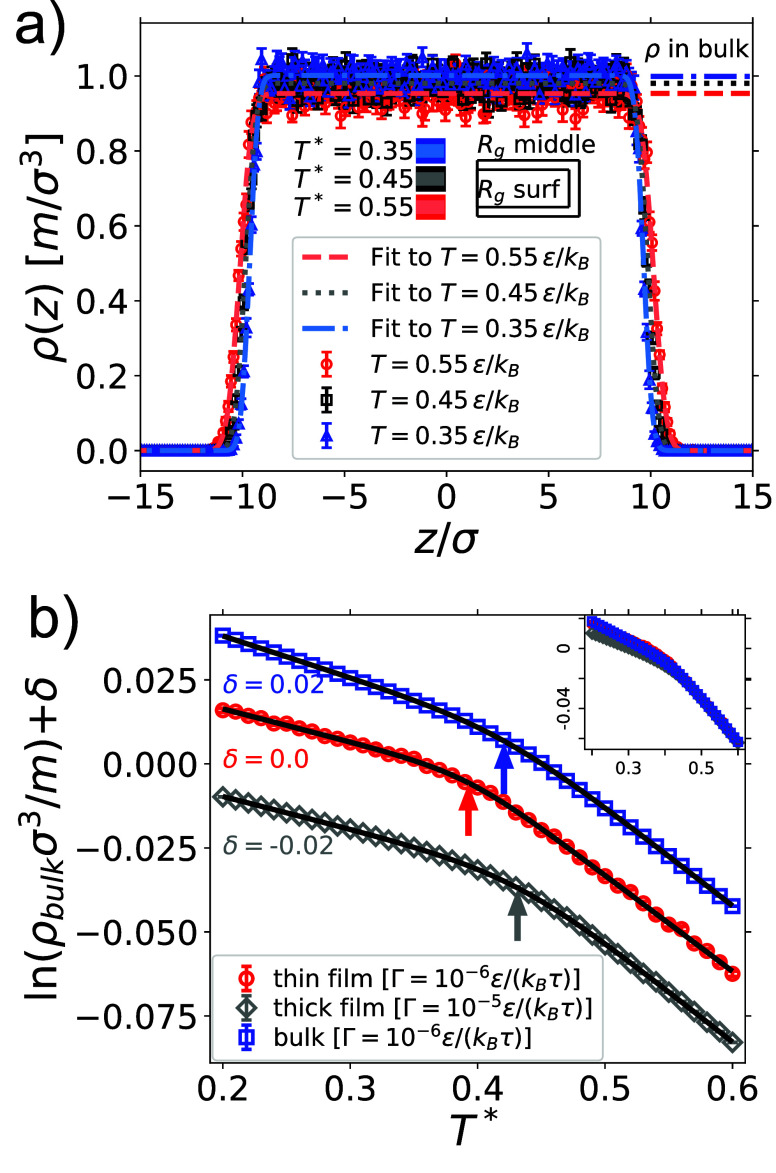
Analysis of the single polymer film and bulk polymer melt subject to constant rate quenching at fixed pressure. (a) Density ρ­(*z*) of the film for different temperatures, calculated at a resolution Δ*z* = 0.125 σ. Data was fit (dashed, dotted, and dash-dotted lines) using eq 1 with two free parameters, ρ_*bulk*_ and δ. The lengths (parallel to the *x*-axis) of the rectangles in the middle of the figure are proportional to the radius of gyration in the middle of the film (outer rectangle, computed for all polymers α such that |*Z*_g,α_| < 2σ where *Z*_g,α_ is the *z*-component of the center of mass of the polymer) and close to the boundary (inner rectangle, computed for polymers whose center of mass is such that *Z*_g,α_ > max_α_(*Z*_g,α_) – 1.5σ or *Z*_g,α_ < min_α_(*Z*_*g*,α_) + 1.5σ) at the listed temperatures. The empty rectangles serve as a legend. The density from bulk simulations [see panel (b)] are shown as lines in the top right corner, using the same color code. (b) Logarithm of the bulk density (blue squares), of the thin film’s bulk density (red circles), and of the thick film’s bulk density (gray diamonds). Error bars represent the standard error on the mean. For clarity, the results for the bulk melt are shifted upward by δ = 0.02, and for the thick film by δ = −0.02. The inset shows the data without the shift. The black lines are fits to eq 2. The arrows show *T*_g_. The most interesting parameters are in Table 2. Uncertainties of data values are commensurate with or smaller than the size of the data points.

**Table 2:** Glass Transition Temperature and Volume Thermal Expansion Coefficients Obtained from the Fit of the Density vs Temperature (Figure 2c)^a^

	bulk [Γ = 10^–6^ϵ/(*k*_B_τ)]	thin film [Γ = 10^–6^ϵ/(*k*_B_τ)]	thick film [Γ = 10^–5^ϵ/(*k*_B_τ)]
*T*_g_ [ϵ/*k*_B_]	0.421 ± 0.001	0.393 ± 0.002	0.4313 ± 0.0009
α_melt_ [*k*_B_/ϵ]	0.296 ± 0.001	0.286 ± 0.003	0.300 ± 0.002
α_glass_ [*k_B_*/ϵ]	0.123 ± 0.002	0.098 ± 0.003	0.0971 ± 0.0007
α_melt_/α_glass_	2.40 ± 0.03	2.92 ± 0.09	3.09 ± 0.03

a The precise value of the fitted parameters slightly depends on the procedure, however the small differences do not affect the conclusions. The results shown are for bulk melt, thin polymer film, and thick polymer film. In parentheses we report the cooling rates.

To extract the dilatometric glass transition temperature, we fit the density to

2 $$\ln\left(\frac{\rho \left(T\right)}{\rho \left(T_{g}\right)}\right)=\frac{w}{2}\left(\alpha_{\mathrm{melt}}-\alpha_{\mathrm{glass}}\right)\ln\left\{\cos h\frac{T-T_{g}}{w}\right\}+\frac{1}{2}\left(\alpha_{\mathrm{melt}}+\alpha_{\mathrm{glass}}\right)\left(T-T_{g}\right)$$

which assumes a jump of the thermal expansion coefficient at *T*_g_ ^31^ (Figure 2). From the fit we get *T*_g_ = (0.393 ± 0.002)­ϵ/*k*_B_ for the thin film. In bulk, the glass transition temperature is slightly larger, *T*_g_ = (0.421 ± 0.001)­ϵ/*k*_B_, in qualitative agreement with previous experimental^32^ and computational^19,33^ measurements for free-standing, thin polymer films. The thick film cannot be used for this comparison because of its faster cooling rate.

To rationalize the time scales of polymer dynamics, we extract the relaxation time of the polymers in the film and in bulk at various temperatures *T* > *T*_g_. For the films, the time-scale for polymer relaxation is faster close to the surface than in bulk.^34,35^ As a probe of polymer relaxation, we investigated the autocorrelation function ϕ­(*t*) of the end-to-end distance,^9^ given by

3 $$\phi \left(t\right)=\frac{8}{\pi^{2}}\sum_{p=1,3,5,\cdot \cdot \cdot }\frac{1}{p^{2}}e^{-p^{2}t/\tau_{R}}$$

where τ_R_ is the Rouse time. For ideal chains, Rouse dynamics provides an exact description of ϕ­(*t*) which depends on temperature and viscosity through the Rouse time τ_R_.^9^ We computed ϕ­(*t*) for polymers in bulk, in the middle of a thin film, and close to the surfaces of a thin film, and fit the data to eq 3 (we include 100 terms in the sum) to establish τ_R_ for each scenario. As shown in Figure 3, all data fall on top of a master curve when scaled by the Rouse time. Close inspection indicates that larger deviations can be seen for polymers at the surface of the films (diamonds in Figure 3b). This is expected; Rouse dynamics assumes isotropy, which is broken near the interface. This analysis allows us to extract the temperature and spatial dependence of Rouse times in polymer films. As shown in the inset of Figure 3b, lowering the temperature increases τ_R_ rapidly in bulk simulations (circles) in the middle of a film (squares) and on the surface of the film (diamonds). However, while the increase of τ_R_ in bulk and in the middle of the film are nearly identical, τ_R_ at the surface is much smaller, indicating that the dynamics of the polymers at the surface is faster than in bulk. In addition, the difference between τ_R_ in bulk and in the surface of a film increases as the temperature is lowered. In Figure S10 of the Supporting Information, we show the logarithm of the Rouse time as a function of the inverse temperature. The curve should be a straight line for Arrhenius-like behavior (τ_R_ ∼ *e* ^*A*/*T*^). For the polymers in bulk and in the middle of the film, it is very clear that the curve is not linear, and a Vogel–Fulcher–Tamman equation (τ ∼ *e* ^*A*/(*T*–*T*_0_)^) is more accurate, as common for glassy systems.

**Figure 3:**
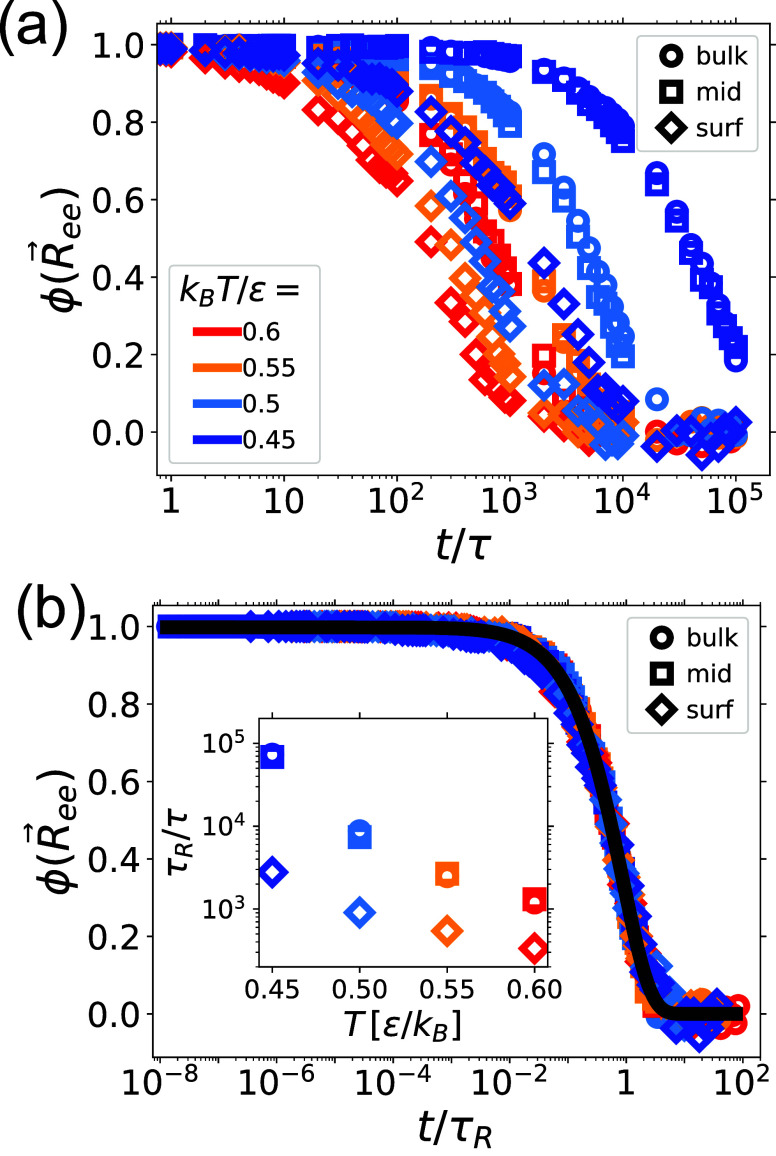
Polymer dynamics in a single film as a function of temperature; colors refer to temperatures noted in the inset of each subfigure. (a) Autocorrelation function of the end-to-end distance for polymers in the bulk melt (diamonds) in the middle of the film (squares, where the selected polymers have |*Z*_*g*,α_(*t* = 0)|< σ) and close to the interface (circles, where surface regions have thickness Δ*z*≈ 1–1.5σ chosen so that about 5% of all polymers are at the surface at all temperatures). (b) We fit the data in panel (a) to the Rouse-chain end-to-end distance autocorrelation function ϕ­(*R⃗*_*ee*_), Equation 3 to extract τ_R_, and scale the time axis by τ_R_(*T*). Inset: Rouse time τ_R_(*T*) as a function of temperature in different regions of the film.

### 3.2 Weld Formation

In the following sections, unless otherwise stated, we focus on thin films. Thick films will be discussed only in Section 3.3

#### 3.2.1 Initial Structure

The setup of the double layer simulation is shown in Figure 1b. From Figure 2b, we selected a low temperature *T*_c_ = 0.35ϵ/*k*_B_, which is lower than our estimated dilatometric bulk *T*_g_ (see Table 2). For the higher temperature we selected *T*_h_ = 0.55ϵ/*k*_B_, with an initial temperature difference across the interface of Δ*T* = 0.2ϵ/*k*_B_. Using the approximate conversion in Table 1, this would corresponds to Δ*T* ≈ 162 K, in line with the temperature gap between ambient and nozzle used in some 3D printing experiments (about 140 K^1^). Note that the dynamics of the polymers in the two films are treated using an energy-conserving algorithm, so that there is no thermal coupling other than with the fluid particles, if they are present. This allows us to monitor the thermal relaxation to equilibrium of the two films via heat exchange across their interface.

#### 3.2.2 Welding Order Parameter

In order to monitor the bonding between the two films, we introduce the welding “order parameter” OP­(*t*) ≡ [*z*_p+1_(*t*) – *z*_p_(*t*)]/σ, proportional to the separation of the welding surfaces. Here, *z̅*_p_ and *z̅*_p+1_ refer to the location of the surface of the bottom and top layer, respectively, which is computed as the spatial average of the monomers at the surface (see Figure 4a and Appendix[Sec app2-sec14]). The results for OP are shown in Figure 4b, from which we distinguish three stages of welding: (I) surface approach up to *t* = 20τ, (II) surface adjustment until *t* = 5 × 10^4^τ, and finally (III) interdiffusion.

**Figure 4:**
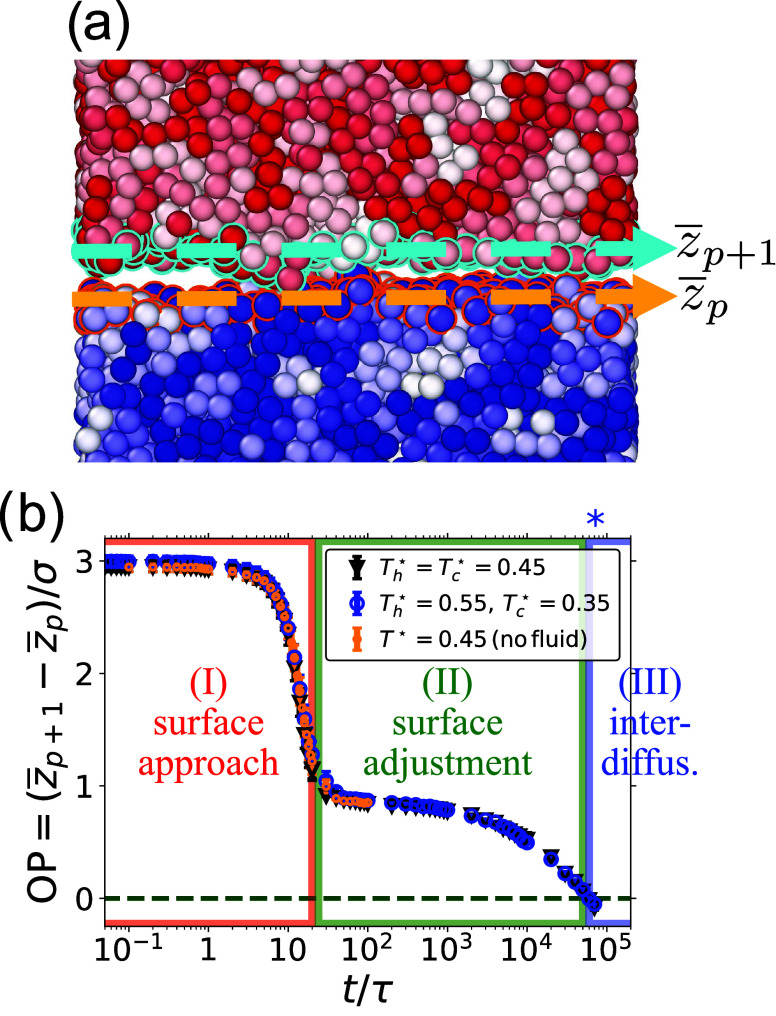
Welding order parameter OP, defined as the normalized separation between the welding surfaces. (a) Pictorial representation of OP. The location of the surfaces of the top (*p* + 1) and bottom (*p*) films are defined as the average positions *z̅*_*p*+1_ and *z̅*_*p*_ of monomers at the surface which are circled in cyan (top) and orange (bottom). These monomers are identified as the beads that are exposed to small probe particles tapping the surface (see Section B.3). Note that the polymers in the top layer are colored in shades from white to red, and those in the bottom layer from white to blue. The precise color is determined by the internal polymer number, and the whole polymer is colored with a single shade, which helps convey the size of the polymers. (b) We define OP­(*t*) ≡ [*z̅*_*p*+1_ – *z̅*_*p*_]/σ. Blue circles refer to the simulation performed by preparing the two films at different temperatures; black triangles denote control simulations in which the two films were prepared at the same temperature; while orange dots refer to control simulations in which the films are in vacuum, without the surrounding fluid. Here we show only the first 100 τ of the simulations in vacuum as we focus on the transition between surface approach and surface adjustment. See the black triangles in Figure S9d for the whole trajectory. The three stages of welding are (I) surface approach (semitransparent red, *t* < 20τ); (II) surface adjustment (semitransparent green, up to 20τ ≤ *t* ≤ 5 × 10^4^τ); (III) interdiffusion (in semitransparent blue, *t* > 5 × 10^4^τ). Recall that *R*_g_≈ 1.45σ. The blue star above the figure indicates the Rouse time τ_R_ at *T* = 0.45ϵ/*k*_B_.

#### 3.2.3 (I) Surface Approach

Initially, OP ≈ 3, so the surfaces are close but not in contact. Within about 20τ the order parameter decreases and it stops at ≈ 1, which we surmise is due to the volume exclusion of the beads at the interface. Consequently, the gap between the surfaces disappears (see the density as a function of time in Figures S4–S5). Control simulations in which the two films were prepared at the same temperature, *T* = 0.45ϵ/*k*_B_, show the same time-dependence OP­(*t*), suggesting OP is insensitive to the temperature difference in this simulation setup. What drives surface approach? Initially, we hypothesized that the external pressure from the fluid might play a role in closing the gap between the two layers. To test this, we rerun the simulations in vacuum and find no significant change over a time scale of <100τ (see orange dots in Figure 4b), although a small discrepancy at around *t* = 20–100τ can be observed for simulations conducted in the presence of the temperature gap at the interface (see Figure S9a,d). This suggests that the films stick to each other due to surface interactions rather than imposed pressure.

Figure 5a shows the temperature of the surfaces of the hot (red, *L*_p+1_) and cold (blue, *L*_p_) films as a function of time. During surface approach, the temperature of the hot film is constant, while at the end of surface approach, the temperature of cold film surface has increased by Δ*T*_c_ ≈ 0.1ϵ/*k*_B_. We rationalize this as follows: if an amount of heat Δ*Q* is exchanged across the interface, the temperature changes of the glass (Δ*T*_glass_) and melt (Δ*T*_melt_) are related by

4 $$C_{\mathrm{glass}}\Delta T_{\mathrm{glass}}=-C_{\mathrm{melt}}\Delta T_{\mathrm{melt}}$$

where *C*_glass_ and *C*_melt_ are the heat capacities of the glass-like and melt-like systems. Because *C*_melt_ > *C*_glass_ ^36^ (similarly the thermal diffusivity increases below *T*_g_ ^23^), a feature that is recovered by our model as shown in Figure S3, we expect |Δ*T*_glass_|> |Δ*T*_melt_|, which agrees with our observations. However, this leads to roughly Δ*T*_h_ ≈ 0.07ϵ/*k*_B_, which is larger than what is observed. Although part of the discrepancy might be due to approximations in our calculation, it is possible that another factor might be involved in determining the thermal evolution of the two surfaces. During control simulations performed by preparing the two films at the same temperature (*T* = 0.45ϵ/*k*_B_), at the end of surface approach the temperature of both surfaces increases by ≈0.05ϵ/*k*_B_ (black and gray in Figure 5a). We surmise that this increase of the local temperature field is due to the surface–surface interaction potential: as the films get closer the potential energy decreases, which results in an increase of kinetic energy and thus of temperature. This effect should be present also when there is a temperature jump between the two films, and thus it should contribute to explaining why the temperature of the *L*_p+1_ (initially hot) layer has not changed at the beginning of phase (I), while the temperature of *L*_p_ (initially cold) has substantially increased.

**Figure 5:**
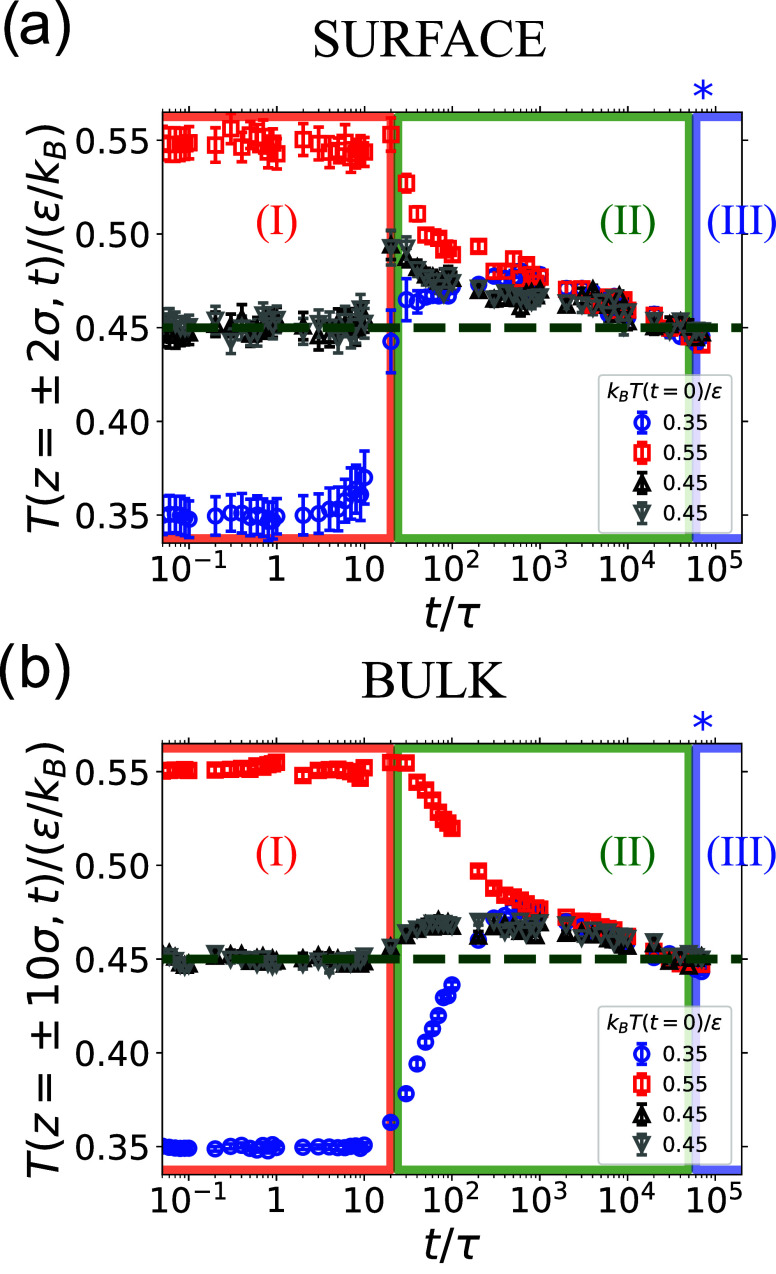
Temperature at the surface and in bulk as a function of time. (a) Temperature computed within 2σ of the hot (*p* + 1, red squares) and cold (*p*, blue circles) surfaces at the interface between films. Black and gray triangles show the results when the two films are prepared at the same temperature, *T*_*m*_ = 0.45ϵ/*k*_B_. The green line dashed line is (*T*_*h*_ + *T*_c_)/2 = *T*_*m*_ = 0.45ϵ/*k*_B_. (b) Same as (a), but with the temperature computed in bulk, that is averaged over three slabs of thickness Δ*z* = 2σ and centered at *z* = 8σ, 10σ, 12σ for the red squares, and around *z* = −8σ, −10σ, −12σ for the blue circles. The three stages of welding (I, II, III) are as in Figure 4b. In both panels, the blue star indicates the Rouse time, τ_R_, at *T* = 0.45ϵ/*k*_B_.

In bulk, far from the interface, the temperature of the two films is nearly the same throughout the surface approach phase regardless of whether there is or not a temperature difference between the two films (see Figure 5b). This is likely because thermal diffusion has not propagated sufficiently far during 20τ.

During the approach phase the thermal gradient influences the structure of the two surfaces at the interface between the two films. Figure 6a shows that the surface of the hot film (red squares) becomes rougher during surface approach, whereas the roughness of the cold film (blue circles) is nearly constant. Right at the end of surface approach, when the two films come into contact, the difference in the roughness of the two surfaces nearly disappears. What drives the roughening of the hot surface? First, we hypothesized that the lack of fluid between the juxtaposed interfaces brings the pressure from *P* = 10^–2^ϵ/σ^3^ to *P* = 0, and as a result the film might expand. Alternatively, surface–surface interactions could increase the roughness of the hot layer, which is in the melt state and thus expected to be softer than the glass film. To test which mechanism is dominant, we simulated the hot film in vacuum (orange dots in Figure 6a): if we observe an increase of Δ_RMSD_ within the same time scale as in our welding simulations (*t* = 20τ), that means that the reduction of the external pressure contributes to increasing interfacial roughness; in contrast, if the surface interactions drive the roughening of the interface, then we should see no change in Δ_RMSD_ in the control simulation. The orange dots show the result of a simulation conducted for a single film in vacuum, and show that in this case surface roughness is constant (Figure 6a), indicating that the second scenario is more likely to be correct, and the hot layer increases its roughness due to surface interactions. In control simulations in which both films are prepared at the same temperature (black and gray triangles in Figure 6a), the initial roughness is intermediate between that of the hot and cold films, and just before the end of the surface approach phase it appears to slightly increase before decreasing upon contact at *t* ≈ 20τ. This supports the idea that surface–surface interactions drive surface roughening, and that this effect is stronger at higher temperatures where the dynamics of the polymers is faster (see Figure 2) and the material is expected to be more pliable.

**Figure 6:**
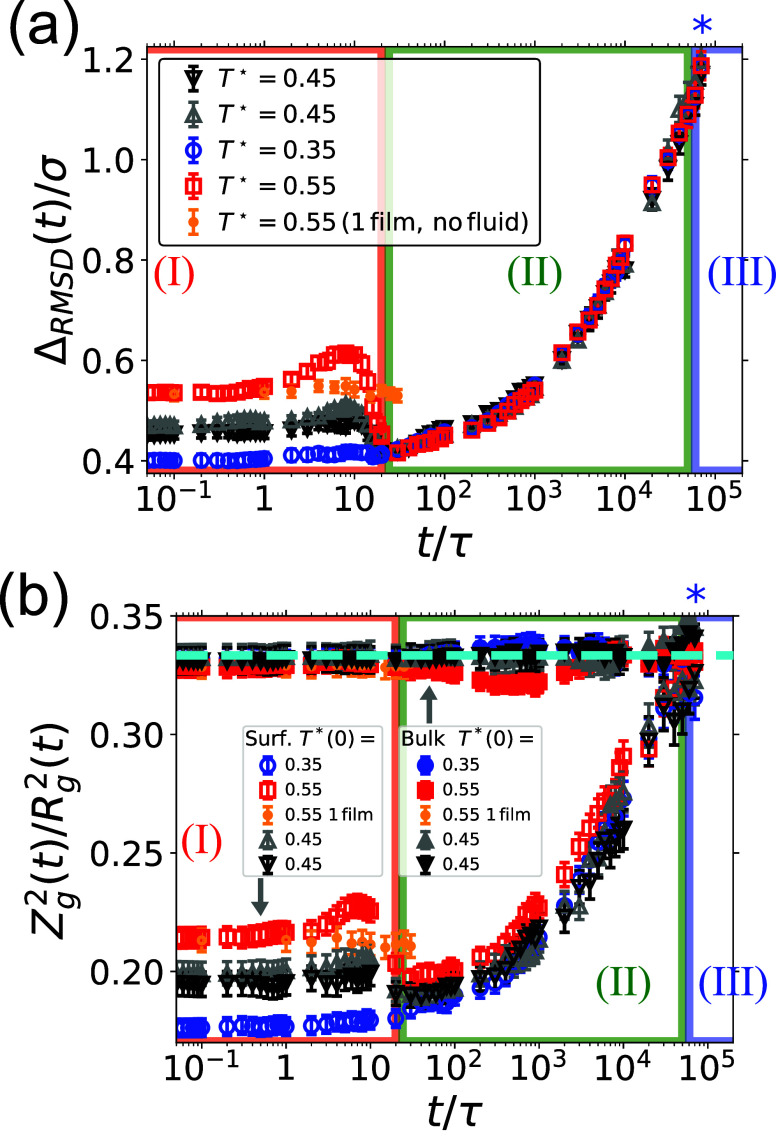
Surface roughness and polymer conformation. In both (a) and (b) the red squares (top, hot, *L*_p+1_) and blue circles (bottom, cold, *L*_p_) are for two juxtaposed films prepared at different temperatures, whereas black and gray triangles are for control simulations in which the films have the same initial temperature (see legend). Orange dots correspond to an adiabatic single film in vacuum without the surrounding fluid to absorb heat at *T* = 0.55ϵ/*k*_B_. (a) Surface roughness as a function of time. (b) Conformation of the polymers at the surface and in bulk, quantified by the ratio of the squared *z*-component of the polymer radius of gyration to the entire squared radius of gyration, for surface or bulk polymers. Surface polymers have centers of mass between −2.25σ and 2.25σ at *t* = 0 (empty symbols), while bulk polymers (full symbols) have centers of mass at *t* = 0 between ± 8.5σ and ± 12.5σ The cyan dashed line shows the value 1/3, which is expected in bulk. In both panels, the background color boxes indicate the three stages of welding (see Figure 4 for details), and the blue star refers to the Rouse time τ_R_ at *T* = 0.45ϵ/*k*_B_.

Figure 6b shows *Z*_*g*_^2^/*R*_g_^2^, where *Z*_g_ is the *z* component of the radius of gyration and *R*_g_ is the total radius of gyration, of polymer in the bulk or near the surface. We expect *Z*_g_^2^/*R*_g_^2^ = 1/3 in an isotropic environment, and indeed in bulk (full symbols) we find *Z*_g_^2^/*R*_g_^2^≈ 1/3 at *t* = 0τ regardless of the temperature, and nearly constant throughout the surface approach. Near the surface (empty symbols), this ratio is smaller than 1/3, suggesting that surface polymers are partially flattened against the interface. Similar to the surface roughness, the *z* projection of the polymer size in the hot film (red, empty squares) increases during the surface approach phase, whereas changes in the conformation of the polymers in the cold film (blue, empty circles) are less significant. Again, the results for an isolated film show no changes in polymer conformations (orange, empty dots), suggesting again that surface interactions lead to polymer adjustments in the hot surface. At the end of the surface approach phase, when the two surfaces are in contact, the *z* projections of the surface polymers in the top and bottom layers nearly match. In control simulations where both films are prepared at the same temperature (gray and black triangles), the *z* projection *Z*_g_^2^/*R*_g_^2^ of the polymers at the surface increases slightly, suggesting again that surface interactions slightly deform the polymers, before *Z*_g_^2^/*R*_g_^2^ decreases upon surface contact to a value slightly below the initial one.

#### 3.2.4 (II) Surface Adjustment

The order parameter OP­(*t*) slowly decays from OP­(*t* ≈ 20)≈ 1 to OP­(*t*) ≈ 0 at *t* ≈ 6 × 10^4^τ (Figure 4b). Because OP > 0 at this point, interdiffusion has not begun, yet. Instead, the films are adjusting to the new interface created upon contact with the juxtaposed film. Figure 5a,b reveal that during this phase, first at the interface and then in bulk, the *L*_p+1_ (initially hot, red squares) and *L*_p_ (initially cold, blue circles) films reach the same temperature, which exceed the average temperature *T̅* ≡ 0.45ϵ/*k*_B_ of the two layers. Next, the two films dissipate energy to the thermalized surrounding fluids and approach *T̅* = 0.45ϵ/*k*_B_ at the end of the surface adjustment phase. The comparison with the control simulations in which the two films were prepared at the same initial temperature (black and gray triangles) reveals that after the initially hot and cold layers have reached the same temperature, the relaxation toward *T̅* = 0.45ϵ/*k*_B_ is independent of thermal history.

Surface roughness increases during surface readjustment up to Δ_RMSD_ ≈ 1.1σ at around *t* = 6 × 10^4^τ when OP ≈ 0 and interdiffusion begins (see Figure 6a). This observation is essentially independent of thermal history; the roughness in the *L*_p+1_ (initially hot, red squares) and *L*_p_ (initially cold, blue circles) films overlap with that for control simulations where the two films where prepared at the same initial temperature (black and gray triangles).

The *z* projection of the squared radius of gyration of polymers at the surface of the two films increases toward (*Z*_g_/*R*_g_)^2^≈ 1/3, suggesting that they “forget” the presence of the surface and relax toward bulk-like, isotropic conformations, which are nearly attained at the end of the surface adjustment phase. As compared to control simulations (black and gray empty triangles) or with the initially cold layer (*L*_p_, blue empty circles), the conformation of the polymers in the initially hot layer (*L*_p+1_, red empty squares) isotropize slightly more rapidly.

The conformation of polymers away from the interface also appears to be influenced by the initial thermal jump across the interface (see Figure 6b). In control simulations, *Z*_*g*_^2^/*R*_g_^2^ ≈ 1/3 in bulk throughout the surface adjustment phase (gray and black full triangles). During welding of films prepared at different temperatures, *Z*_g_^2^/*R*_g_^2^ for *L*_p+1_ (initially hot) polymers initially in bulk (red full squares) slightly decreases between *t* ≈ 100τ and 2000τ, and correspondingly we observe a weak increase of *Z*_g_^2^/*R*_g_^2^ for the bulk *L*_p_ (initially cold) polymers (blue full circles). We rationalize this observation as follows. As the high-temperature polymer film cools its density increases. Because the area of the film is fixed, the film becomes thinner (*L*_*z*,p+1,melt_ decreases). It is reasonable to imagine that this uniaxial compression would affect the polymers as well, and indeed this is reflected in *Z*_g_^2^/*R*_*g*_^2^ < 1/3 at the bulk at these times. To quantitatively test this observation, we note that at *t* = 700τ, the temperature of *L*_p+1_ (and *L*_p_) is *T*_*x*_ ≈ 0.48ϵ/*k*_B_. Using the thermal expansion coefficient α computed from single-film simulations (Figure 2b) we predict a polymer contraction for polymers in *L*_p+1_ given by

$$\begin{array}{c} \frac{Z_{g,\mathrm{melt}}^{2}\left(T_{x}\right)}{R_{g,\mathrm{melt}}^{2}\left(T_{x}\right)}\approx \frac{{\left[1+\alpha_{\mathrm{melt}}\left(T_{x}-T_{h}\right)\right]}^{2}}{2+{\left[1+\alpha_{\mathrm{melt}}\left(T_{x}-T_{h}\right)\right]}^{2}} \end{array}\approx 0.324$$

in excellent agreement with our simulations (Figure 6b), which give 0.323 ± 0.004. Following the same logic, one should expect an increase in *Z*_g_^2^/*R*_g_^2^ for the cold layer, which should be weaker because the cold layer has a smaller thermal expansion coefficient and thus its density changes less than the hot film (Figure 2b and Table 2). In this case, though, the thermal expansion coefficient changes rapidly across *T*_g_, and thus a quantitative calculation for *L*_p_ analogous to what that for *L*_p+1_ seems too simplistic.

#### 3.2.5 (III) Interdiffusion

We interpret the beginning of interdiffusion to occur for OP­(*t*) ≤ 0, which starts at *t* ≈ 6 × 10^4^τ (Figure 4b), which means that the two surfaces have crossed each other. At this point, the temperature jump across the interface has dissipated (Figure 5) and the polymers that initially were in bulk and at the surfaces have attained isotropic conformations (Figure 6b). At this stage, for this model, it is then reasonable to expect that thermal history does not have a significant impact on the interdiffusion stage of welding.

What sets the time scale of the onset of interdiffusion? I.e. why is OP ≈ 0 for *t* ≈ 6 × 10^4^τ? To answer this question we prepared two juxtaposed films at the same temperature *T*_m_, with *k*_B_ *T*_m_/ϵ = 0.5, 0.55, and 0.6. We focus on the trajectories after initial contact is formed, which means in practice that we neglect the first 100τ. Figure 7a shows |OP­(*t*/τ_R_)|, where we have scaled time by the polymer bulk Rouse time τ_R_≈ 7 × 10^4^τ at the average temperature *T̅*≈ 0.45ϵ/*k*_B_ between the two films (see inset of Figure 3b). The data fall onto a master curve with a cusp at *t*/τ_R_ ≈ 1, suggesting that the onset of interdiffusion (that is, OP = 0) occurs at around bulk τ_R_. Similarly, we monitor *Z*_*g*_^2^/*R*_g_^2^ for polymers initially at the vicinity of the interface between the two juxtaposed films. Figure 7b shows that if we scale time with the Rouse time at the intermediate temperature between the two films, we see that the emerging master curve indicates that *Z*_g_^2^/*R*_g_^2^ ≈ 1/3 at *t* ≈ τ_R_; hence the Rouse time τ_R_ controls how long it takes for surface polymers to “forget” the presence of the interface, which is indeed the simplest explanation. In the case of *Z*_g_^2^/*R*_g_^2^, the collapse of the data onto a master curve is less successful, partially owing to noise, and perhaps because the Rouse time for polymers at the surface is smaller than for those in bulk (see Figure 2b). As shown in Figure 7a, the welding order parameter OP­(*t*) grows nearly linearly in time for *t* > τ_R_ (its standard deviation grows instead as *t* ^1/3^, see Figure S6). Small deviations from this scaling appear at the largest time, possibly due to finite-size effects, when polymers reached the opposite side of the juxtaposed film.

**Figure 7:**
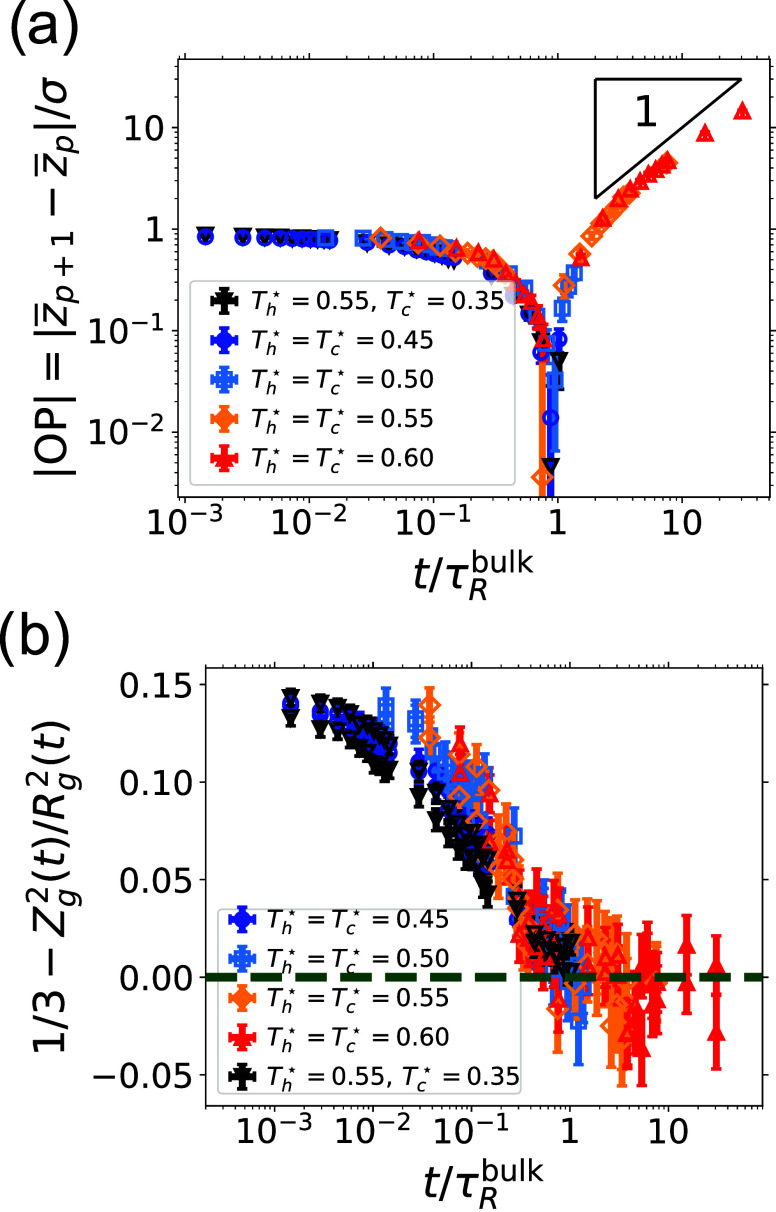
Long-time dynamics at the interface between two layers. In all panels, black downward triangles refer to simulations conducted by juxtaposing two films prepared at different temperatures. Blue circles, light blue squares, orange diamonds, and red upward-facing triangles refer to simulations performed by juxtaposing two layers prepared at the same temperature, respectively *T* ^*^ = 0.45, = 0.50, = 0.55, and = 0.60. Time is scaled by the bulk Rouse time, obtained as in Figure 3. (a) Evolution of the absolute value of the order parameter OP­(*t*). The triangle in the top-right corner indicates a slope of 1. (b) The anisotropy of the polymer conformation, quantified by the deviation from 1/3 of the square of the *z*-component of the radius of gyration relative to the square of the total radius of gyration for polymers initially close to the interface (see Figure 6 for details). The dashed green line indicates 0, which is expected for an isotropic system.

### 3.3 Energy Dissipation

The fluid surrounding the polymer melts is thermostated and maintained at its initial temperature, so it absorbs or releases heat associated with weld formation (see Figure 1b). This results in a nonequilibrium system whose overall energy and temperature (see Figure S7) depend on time until they become stationary. This mimics the scenario during fused filament fabrication, where energy is dissipated to the environment and to other previously deposited layers. It is instructive to test how the system would behave in the absence of thermalization. Hence, we uncoupled the fluid from the thermostat and repeated the simulations. As shown in Figure S8, for which the energy is conserved, the temperature increases to *T*≈ 0.47ϵ/*k*_B_, larger than the average temperature *T*_m_ = (*T*_h_ + *T*_c_)/2 = 0.45ϵ/*k*_B_ between the two films at *t* = 0. Interestingly, as shown in Figure S9a–c, the OP reaches 0 faster without thermalization than in the presence of thermal coupling, similarly it displays faster-increasing roughness and quicker isotropization of the surface polymers. As a further test, after juxtaposing the two films we remove the fluid and run simulations in vacuum, which we can compare to the welding of the thick polymer films. The results in Figure S9d reveal two interesting findings. First, despite the fact that the OP monitors surface properties, a thicker film has a longer delay before the onset of interdiffusion. Second, at given film thickness, the existence of a temperature jump at the interface between the two films at time *t* = 0 leads to faster onset of interdiffusion than if there was no temperature jump. In order to interpret these observations, we note that diffusion begins earlier for films at higher temperature (see Figure 7a) On the basis of this observation, we draw the following two conclusions: (i) simulations reveal that thicker films are colder than thin ones, as shown in in Figure S9e,f. We surmise that this is due to the following: as the surface energy released during welding dissipates through the system it increases the system’s temperature. However, the surface energy is proportional to the interfacial area, whereas the increase in temperature depends on the whole volume of the system. Hence, thicker films are expected to be colder. (ii) At given thickness, in the absence of energy dissipation to the environment, the temperature is higher if the system is prepared in the presence of a thermal gradient, as revealed in Figure S9e,f. This is likely a consequence of the fact that the polymer melt has larger specific heat than the polymer glass. These observations point to a long-time effect of the temperature difference. Of course preparing an isothermal set of two films at higher temperatures, so that the final equilibration temperature is the same as in the case in which there is temperature difference between the two films before welding, would recover this behavior, but the comparison is incongruous because it provides the isothermal system with overall higher energy.

## 4 Discussion

In this study, inspired by FFF, we have investigated the effect of the temperature difference on the welding of two juxtaposed polymer films. We first prepared the polymer films at different temperatures by cooling them at a given rate, and used these simulations to estimate the dilatometric glass transition temperature. Next, we juxtaposed two films either prepared at different temperatures or at the same temperature and monitor the formation of the weld, and we highlighted the importance of surface interaction and of the presence of a thermal gradient at the interface. Finally, we conducted simulations at higher temperature to explore the long-time dynamics across the interface, and we tested whether insulating the system by removing the fluid and changing film size impact welding.

### 4.1 Single Film Glass Transition Temperature

The thickness of the film that we simulate is expected to affect the glass transition temperature, as shown in various experimental^32^ and computational^19,37^ studies. Our results for the change in thermal expansion coefficient (Figure 2a) with temperature agree with this observation: the glass transition temperature of the film surrounded by a fluid is lower than bulk *T*_g_. This suggests that the polymers at the interface behave differently from those in bulk. This agrees with our results for the dynamics of polymers and monomers in the vicinity of the surface or in the middle of a thin film (see insets in Figure 3a,b). Other simulations^29,38^ and experiments^35,39^ have illustrated the different dynamics at the interface and in the bulk, and the concept has been used to design ultrastable glasses.^39,40^ Finally, the layer model used to explain the dependence of *T*_g_ on film thickness posits the existence of a surface and a bulk glass transition temperature.^34^ Our observations correlate with this model. Note that the thick and thin films are cooled at different rates, and thus the discrepancy in their glass transition temperature cannot be ascribed exclusively to system size.

### 4.2 Stages of Welding

Our simulations enabled us to identify three initial steps during the welding process. First, the surfaces approach each other and reach contact, which occurs at around *t* ≈ 20τ. Second, a long phase of surface readjustment begins, in which the two interfaces have not crossed yet. Third, at around the Rouse time, the interfaces cross and interdiffusion begins. These steps mirror the stages of crack healing identified by Wool and O’Connor,^6^ namely (i) approach of the two surfaces, (ii) wetting, and (iii) diffusion across the interface.

#### 4.2.1 Surface Approach and Initial Contact

During the surface approach phase, we find five principal observations.

(i) Upon contact, the two surfaces are at a distance OP­(*t* = 0) ≈ 3, which is determined by the occurrence of two protrusions below the top and above the bottom surface that come into contact. This distance is controlled by the roughness of the film, which is described by capillary waves.^41^ Had we considered a larger film, both the likelihood of contact and the roughness would have increased, which means that the initial value of the order parameter would have likely been >3σ. It seems reasonable that in this case, the initial approach phase would last somewhat longer, as it would take more time to draw the two surfaces in contact. After that, we surmise that the following stages of welding would proceed as we described them here, but systematic studies are necessary in order to ascertain this hypothesis.
(ii) The closure of the initial gap between the two films is driven by surface interactions. We have ignored gravity, which is irrelevant in our system given the small thickness of the film, but could play a role during 3D printing. To ascertain whether our approximation is reasonable, we compare the compression between two cylinders kept at distance *D* due to gravity force with the van der Waals (vdW) interaction. The gravity force per unit length is *F*_g_/*L* = π*R* ^2^ρ*g*, whereas the vdW force is given by *F*_vdW_/*L* = *AR* ^1/2^/(16*D* ^5/2^) (see Figure 13.1 in ref 42, where the two cylinders are assumed to have the same radius), where *R* is the radius of the cylinder, ρ is the mass density, *g* is the gravitation acceleration, and *A* is the Hamaker constant. For *D* < *D* ^*^ = [*A*/(16πρ*gR* ^3/2^)]^2/5^ vdW forces dominate. We estimate ρ ≈ 1 g/cm^3^, *R* ≈ 0.2 mm (see Figure 7 in ref 2), and *A* ≈ 10^–19^ J,^42^ to obtain *D* ^*^ ≈ 22 nm. At distances commensurate with the initial separation between two films in our simulations, that is, a few nanometers, vdW forces should be at least 1 order of magnitude larger than gravitational forces.
(iii) We infer that surface interactions increase the roughness of the interfaces just before contact (Figure 6a). This is mirrored by the orientation of the polymers at the surface (Figure 6b); the interaction with the other film tends to slightly change the polymer orientation away from spontaneous alignment parallel to the surface. This mechanism might help bridge the two surfaces by breaking up the filament that separates them, and be similar to the second step of droplet coalescence identified by Aarts et al.^43^
(iv) As the interfaces come into contact, surface energy from the interaction potential between the adjoining surfaces at the interface is converted into thermal energy, and locally the temperature rises at the welding site, before diffusing across the whole system (orange curve in Figure 5b). At *t* = 20τ the increase of temperature at the interface is Δ*T* ≈ 0.05ϵ/*k*_B_ (see Figure 5a, black and gray curves). For physically realistic polymers, using ϵ in Table 1 we obtain Δ*T* ≈ 41 K. In principle, techniques such as infrared (IR) thermography^1^ could detect the extent of this predicted localized heating.
(v) Upon contact, the distance between the two surfaces is approximately the same as the diameter of a monomer. We interpret this as a reasonable consequence of volume exclusion.

#### 4.2.2 Surface Adjustment

After initial contact is formed, the thermal gradient dissipates and the temperature in the two films becomes homogeneous within approximately (500–1000)­τ (Figure 5). At around the same time, all of the observables that we monitored [temperature and density fields (Figures 5 and S4–S5), surface roughness (Figure 6a), and polymer orientation at the surface (Figure 6b)] evolve nearly identically to those of juxtaposed films prepared at equal temperatures, and all have reached an equilibrated state by the end of the surface adjustment phase. Four features are noteworthy.

(i) The time dependence of the temperature in the vicinity of the interface and in the middle of the film (Figure 5a,b) resembles the temperature profiles measured for two successively deposited layers using IR thermography (see Figure 7 in ref 1). Of course, the time- and length-scales explored in experiments and simulations are vastly different, yet we recover the observation that the two layers become isothermal at a temperature above the final, equilibrium temperature. This increase is due to at least two factors. First, the difference in heat capacity between the hot and cold layers results in a faster change in temperature of the cold layer compared to the hot layer. Hence, thermal equilibrium would be attained at a temperature higher than the (mass-)­average temperature of the two layers. Second, the conversion of surface potential energy into kinetic energy upon approach to OP≈ 1 should contribute to this phenomenon.
(ii) As the initially hot layer cools down, the film compresses along the *z* direction, which affects the conformation of the bulk polymers, see Figure 6b. Taking polystyrene as an example, and assuming that the temperature of the melt quickly changes by ≈ 50 K, then in the newly deposited layer *L*_p+1_, eq 5 leads to *Z*_g,melt_^2^(*T*_*x*_)/*R*_g,melt_^2^(*T*_*x*_) ≈ 0.32, where the volume thermal expansion coefficient is α_melt_ ≈ 5–6 × 10^4^/K.^22^ This is a small effect and vanishes rapidly as the polymers readjust and heat is transferred to the environment.
(iii) The time scale for thermal relaxation is ∼10^2^–10^3^τ, which is about the same as the time scale for thermal diffusivity. Given that our films are approximately *L*_*z*,melt_ ≈ 20σ thick, the thermal relaxation time scale is about τ_*D*_ ∼ *L*_*z*,melt_^2^/*D*_T_ ≈ 300τ, in reasonable agreement with our MD results.
(iv) The strength of the bonding between the interfaces is expected to grow during this phase because the roughness of the interface increases. Indeed, increased roughening should correlate with a greater amount of interdigitation, resulting in more interactions between the polymers belonging to different films, in analogy with experimental results on adhesion between two polymeric surfaces^44^ and with theories that relate, at least in part, weld thickness with mechanical strength.^4^

#### 4.2.3 Interdiffusion

In our model, Figure 7a shows that interdiffusion begins at ≈ τ_R_^bulk^, the bulk Rouse time of the polymer. This means that by the time that interdiffusion has begun, the polymers at the surface of the two films have had enough time to forget their initial state and attain bulk-like conformations (Figure 7b). For a realistic system, other factors are likely to play a role in determining the start of interdiffusion after fused filament fabrication. (i) The polymers are typically much longer, with reorientation dynamics governed by the much longer disentanglement time τ_d_ ≫ τ_R_.^9^ (ii) When polymers are extruded from the nozzle they are stretched and oriented with the flow, particularly in the vicinity of the weld.^4^ These observations suggest that simulations of longer polymers prepared under shear^45^ will be important to extract the time scale for the beginning of interdiffusion. (iii) The way in which heat is dissipated by the system is also consequential, as we discuss in the next section.

### 4.3 Energy Dissipation and Film Thickness

If the fluid surrounding the two polymer films is uncoupled from a thermostat, the final temperature of the welding layers is larger. This may explain why uncoupling the films from a thermal bath shortens the surface adjustment phase of welding and thus leads to an earlier onset of interdiffusion. Turning off heat transfer is not realistic, but neither is the size of the polymer films (a few nm) compared to layers deposited in 3D printing (∼ 500 μm). In addition, removing the coupling to a thermal reservoir reveals an important observation: the manner in which energy is dissipated affects the progress and timing of welding. This suggests that simulations of larger polymer films would be extremely interesting as a next step in the investigation of the welding in the presence of thermal gradients. To this end, we prepare a polymer film with the same area as those described so far, but 11-times thicker. The film is in vacuum, so at normal pressure *P* = 0ϵ/σ^3^. We compare the results of the thick film with equivalent simulations done for the thin film in which, after creating the juxtaposed pair of films, we removed the surrounding fluid. We find that the onset of interdiffusion is delayed if (i) the system is increased in size, and (ii) at given thickness, if the films are prepared at the same, intermediate temperature. These observations are likely a consequence of the effect of surface energy and specific heat imbalance between the polymer melt and polymer glass.

To summarize, when the two systems rapidly thermalize through contact with the environment, whether or not the films are prepared in the presence of a thermal gradient has little effect on the order parameter that monitors the progression of welding. In contrast, if the system is isolated, the impact is significant. In reality, the deposited material is significantly thicker than our model film, which results in a time-evolving temperature at the interface, and the persistent presence of heat flux between the two films. We note that for our simulation the temperature gap between the two films dissipates in about *t*_*Q*_ ≈ (500–1000)­τ ≈ 5–10 ns, whereas in experiments, where the thickness of the deposited layer is ∼0.3 mm, *t*_Q_ is of the order of 1–3 s.^1^ This suggests that heat transfer continues for a long time across the interface, and it would be interesting to study how it impacts the dynamics of the polymers. Ideally, it would be important to monitor the ratio τ­(*n*)/τ_Q_ between the characteristic time scale of polymer relaxation at the surface, where τ­(*n*)/τ ∼ *n* ^2^ (*n* here is the number of beads of the polymer) dictates the beginning of interdiffusion, and τ_Q_ ∼ *L*_melt_^2^/*D*_T_ is the time for heat to travel through the whole simulated film, where *L*_melt_ is the film thickness and *D*_T_ is the thermal diffusion coefficient. Finally, we should also point out that a more comprehensive model would also account for the heat transfer occurring far from the interface, either to a cold environment, or to the previously deposited layers which are maintained at higher ambient temperature.

### 4.4 Welding and Thermal Transport in Coarse Grained Model

The resolution of our model is of the order of a coarse-grained bead. When the surfaces come into contact, interactions at the atomic scale likely play a role in shaping the larger scale surface–surface interactions. In this sense, it would be instructive to investigate how our results would change by using an all-atom model to study the early stages of welding. We expect that thermal transport in a coarse-grained model might not recapitulate what has been observed in experiments, since many microscopic degrees of freedom (that contribute to dissipation) have been neglected. In order to test the performance of the model, we computed the thermal conductivity and diffusivity and compared them to experimental estimates for atactic polystyrene (refs 22,23 , see Table 1 for details). Whereas our model underestimates thermal conductivity by a factor ≈20, the thermal diffusivity, which relates temporal and spatial changes of the temperature field, appears to be faster in our model by only a factor 3. This suggests that our time scale is not unrealistic, particularly considering that, as has been suggested, the smoother potential in the coarse-grained model compared to the all-atom one gives rise to faster dynamics that can be corrected using appropriate choices for the friction coefficient,^46^ and we did not use a frictional term in our equations of motion to avoid coupling the polymer melt to an external thermostat (such as in case of Langevin dynamics) in order to focus on thermal transfer between films. We should also note that, for *T* > *T*_g_, while in the experiments that we used as reference in this study the specific heat increases with temperature^22^ and the thermal diffusivity slightly decreases or remains nearly constant,^23^ we see the opposite trends in our simulations in the range explored (see Figure S3). These observations suggest that the model could be improved in order to produce a more accurate thermodynamic description of polymer melts. This also is expected given that our polymer model has many fewer degrees of freedom than real polymers.

## 5 Conclusions

Inspired by FFF AM, we performed MD simulations to understand the differences between bonding between films prepared at the same temperature versus welding in the presence of a temperature gap at the interface. In both cases, we identified three stages of welding: (I) surface approach and formation of the initial contact, (II) surface adjustment, and (III) interdiffusion. In simulations in which thin films are surrounded by a fluid that absorbs the heat released during welding, the timing of the stages is not affected by whether the temperature of the films differs. Our major observations are the following:

(i) Surface energy released upon contact results in a local increase of temperature in the two films, which is progressively dissipated.
(ii) The cold film changes its temperature more rapidly than the hot one, presumably due to the smaller heat capacity of polymer glasses compared to melts.
(iii) Compared to simulations in which the welding films were prepared at the same temperature, the initially hot and cold film display differences in the structure and dynamics of the polymers predominantly at the interface, but with some small effects propagating away from it.
(iv) The order parameter monitoring welding signals that regardless of the temperature gap between the two layers at the beginning of the simulations, interdiffusion begins at around the bulk Rouse time.

Hence, isothermal simulations of welding films coupled with thermal reservoirs, as they are commonly done, are extremely interesting to describe the long-time behavior of juxtaposed films even if they had a large temperature gap upon contact. However, if we simulate the films in vacuum, so that they are isolated and cannot dissipate energy in the environment, we find that welding is delayed as the thickness of the film is increased, and that welding occurs more rapidly if there is a temperature gap between the two films at the beginning of the simulation. We surmise that these two observations can be explained by (i) the dissipation of surface energy through the film, which increases more the temperature in small films and thus reduces the waiting time to observe interdiffusion, and (ii) by the fact that melts have a higher specific heat than polymer glasses, resulting in a larger temperature of the two films during welding. This suggests that by selecting a material with a larger difference in specific heat between the glassy and the melt state, it may be possible to speed up the onset of interdiffusion, create a stronger weld, and improve material performance. Further simulations and, more importantly, experiments could be use to corroborate our suggestion and verify our prediction.

## Supplementary Material



## A. Methods

We performed coarse-grained molecular dynamics (MD) simulations using LAMMPS.^16^ Equations of motion are integrated using a time step d*t* = 0.001τ or d*t* = 0.002τ, where τ is the internal unit of time, as described next. Different algorithms are used for the various steps needed to prepare the system.

### A.1 Units

All quantities are shown in Lennard-Jones (LJ) units: lengths are given in units of *σ*, which is roughly the diameter of a monomer (or bead) of the polymer; energies are scaled by ϵ, the depth of the attractive interaction; consequently, the temperature scale is ϵ/*k*_B_, where *k*_B_ is the Boltzmann constant; units of time are $\tau =\sqrt{m\sigma^{2}/\epsilon }$ , where *m* is the mass of a monomer; densities are given in units of *m*/σ^3^, and the pressure scale is ϵ/σ^3^. Dimensionless quantities are denoted by a “*”; for instance, the pressure *P* = *P***ϵ*/σ^3^, where *P** is the dimensionless value of the pressure. Everaers et al.^21^ have provided tables to convert a polymer model (Kremer-Grest) similar to the one that we have used (see below) to commodity polymers. Table 1 contains conversion parameters for polystyrene, in which we selected ϵ in order to match the experimental bulk glass transition temperature in the infinite molecular weight limit, and σ and *m* are chosen so that the Kuhn length and Kuhn mass of the model are identical to the experimental values following a procedure inspired by Everaers et al.^21^ The discrepancy in the experimental and simulated density suggests that the model is not precisely suited for polystyrene; however, the conversion factors can be used to roughly convert from simulation properties to physical properties, such as temperature, molecular weight, and polymer structure.

### A.2 Polymer Model

We use a variant of the polymer model recently introduced by Hsu and Kremer (HK),^18^ which is a modification of the Kremer-Grest (KG) model^20^ in order to study the glass transition in polymer melts. Following HK (and KG), we use the sum of a Finitely Extensible Nonlinear Elastic (FENE) and Weeks–Chandler–Anderson (WCA) potentials for the bonded interaction between consecutive monomers within a polymer. The FENE potential is given by

A1
$$U_{\mathrm{FENE}}\left(r\right)=-\frac{1}{2}KR^{2}\;\ln\left[1-{\left(\frac{r}{R}\right)}^{2}\right]$$

where *K* = 30ϵ/σ^2^ is a spring stiffness, *R* = 1.5σ is the maximum extension allowed, and *r* is the distance between two beads. The WCA potential is a Lennard-Jones potential truncated at the minimum and shifted so that energy and force are continuous, that is,

A2
$$\begin{array}{l} U_{\mathrm{WCA}}\left(r\right)=\left\{4\epsilon \left[{\left(\frac{\sigma }{r}\right)}^{12}-{\left(\frac{\sigma }{r}\right)}^{6}\right]+\epsilon \right\}\Theta \left(r_{\mathrm{cut}1}-r\right)\\ r_{\mathrm{cut}1}=2^{1/6}\sigma \approx 1.12\sigma \end{array}$$

where the step function [Θ­(*x*) = 1 for *x* > 0 and = 0 otherwise] truncates this interaction at the minimum (*r*_cut1_). The non-bonded interaction between monomers of the polymer is given by

A3
$$\begin{array}{l} U_{\mathrm{nb}}\left(r\right)=4\epsilon \left[{\left(\frac{\sigma }{r}\right)}^{12}-{\left(\frac{\sigma }{r}\right)}^{6}\right]\Theta \left(r_{\mathrm{cut}1}-r\right)-\epsilon \cos{\left[\frac{\pi }{2}\frac{\left(r-r_{\mathrm{cut}1}\right)}{\left(r_{\mathrm{cut}2}-r_{\mathrm{cut}1}\right)}\right]}^{2}\left[\Theta \left(r-r_{\mathrm{cut}1}\right)\cdot \Theta \left(r_{\mathrm{cut}2}-r\right)\right]\\ r_{\mathrm{cut}1}=2^{1/6}\sigma \approx 1.12\sigma \\ r_{\mathrm{cut}2}=2^{2/3}\sigma \approx 1.59\sigma \end{array}$$

Here, the first term is a WCA potential without the upwards shift of the energy, and it constitutes the repulsive core of the inter-particle interaction. The second term is a short attractive tail modeled using a cosine function that smoothly goes from −ϵ at the truncation point of the WCA potential to 0 at *r*_cut2_ = 2^2/3^σ ≈ 1.59σ.

Inspired by the HK model^18^, the function *U*_*nb*_ approaches zero smoothly at *r*_cut2_, which is well suited for energy conserving algorithms (Figure S1b). Also the HK model arrives smoothly at 0 at around the same value as our *r*_cut2_, though the functional form is slightly different (see Figure S1, our choice was made only on the basis of convenience in the implementation within LAMMPS, the differences are fairly small). However, the attractive tail is different from the Bennemann polymer glass models^37,47,48^ in which the interaction is described by the standard LJ potential and was truncated at 2 × *r*_cut1_ ≈ 2.24σ (see Figure S1). The structure of the melt is not expected to be significantly affected by the change in the attractive tail, in agreement with the so-called Flory theorem,^49,50^ with analytical models,^9^ and with numerical simulations.^51^ However, as illustrated in simulations comparing models featuring or lacking an attractive tail to the two-body interaction potential,^51^ the dynamics of the polymers is expected to differ, and thus *T*_g_ for the Bennemann and the HK models will not be the same. We consider a flexible chain with no bending rigidity, which differs from the HK model.^18^ Because both the bending rigidity of the chain^52^ and the length of the polymer^24^ are expected to increase *T*_g_, the estimate of *T*_g_ ≈ 0.64ϵ/*k*_B_ in bulk for 50-mers by HK is not expected to hold for our model (see Table 2).

For each thin film, we use *N* = 1000 polymers of length *n* = 10 and mass *mn*. We initially place them in a box at density ρ = 0.85 *m*/σ^3^, with box size *L* ≈ 22.74 σ. For bulk simulations, the volume is controlled by an isotropic barostat and depends on temperature (see below). For simulations of polymer films, the box size in the *x* and *y* direction is fixed, and hence the area of the interface between two films is *A* = *L* ^2^ ≈ (22.74 σ)^2^. The size of the system in the *z* direction depends on its temperature and pressure (see below).

The bulk radius of gyration is given by *R*_*g*_^2^ = ⟨*R*_*g*,*α*_^2^⟩, where *R*_*g*,*α*_^2^ = 1/*n*∑_*i* = 1_^*n*^(*r⃗*_*i*,*α*_ – *r⃗*_*α*_)^2^,^9^ *r⃗*_*i*,*α*_ is the location of the *i*-th bead in the *α*-th polymer, *r⃗*_*α*_ = 1/*n*∑_*i* = 1_^*n*^ *r⃗*_*i*,*α*_ is the center of mass of the *α*-th polymer and ⟨···⟩ here refers to both a time average and an average over all polymers, and the end-to-end distance is *R*_ee_^2^ = ⟨*R*_ee,*α*_^2^⟩, where *R*_*ee*,*α*_^2^ = (*r⃗*_*n*,*α*_ – *r⃗*_1,*α*_)^2^ and ⟨···⟩ refers to both averages over time and over polymers. We find that *R*_*g*_ and *R*_*ee*_ are only weakly dependent on temperature and at *T* = 0.6ϵ/*k*_B_ we find *R*_g_/σ = 1.4530 ± 0.0007, *R*_ee_/σ = (3.527 ± 0.003), with *R*_ee_^2^/*R*_*g*_^2^ = 5.89 ± 0.01, not far from the theoretical expectation for an infinitely long Gaussian chain, *R*_ee_^2^/*R*_*g*_^2^ = 6.^9^ Here, the errors on the mean polymer size are obtained from the time-dependent average radius of gyration in the melt. To take care of the time correlation of the data, we used the following equation to compute the errors:^53^

$$\sigma^{2}({\left\langle x\right\rangle }_{t})=2/t\int_{0}^{t}dt^{\prime }(1-t^{\prime }/t)\left\langle \delta x(t^{\prime })\delta x\left(0\right)\right\rangle$$

where δ*x*(*t*) = *x*(*t*) – ⟨*x*⟩, ⟨*x*⟩_*t*_ = 1/*t*∫_0_^*t*^ d*t*′*x*(*t*′) (time average), and we approximated the integral by truncating it once the argument reaches zero in order to avoid integrating over long, noisy tails.

### A.3 Surrounding Fluid

In order to create an interface, the polymer film has to be equilibrated either in vacuum (free-standing film), rigidly confined by an impenetrable barrier, or in contact with a different fluid. Free-standing films are a valid option which corresponds to a fixed pressure = 0 normal to the interface and end up eliminating an environment that can exchange heat, thus providing an adiabatic boundary condition. Rigid walls alter the structure of the melt near the wall.^48^ Thus, we initially coupled our films to an external fluid, which allows us to control the applied normal pressure and provide thermal coupling to the welding films.

We model the interactions within the fluid and between fluid and polymers using a WCA potential (eq 7, Figure S1) which ensures that the fluid will not undergo liquid-gas or (under the conditions simulated) liquid-solid transitions.^54^ We use *N*_fluid_ = 907 fluid particles per film, and we place them in a box of area *A* ≈ (22.74σ)^2^ at a fixed pressure *P* ≡ *P*_*zz*_ = 0.01ϵ/σ^3^, which allows the thickness *L*_*z*_ to fluctuate. This corresponds to roughly 1 atm (Table 1). The fluid thus obeys an equation of state of the form *L*_z,fluid_ = *L*_z,fluid_(*T* |*P*, *N*, *A*).

The equation of state of a low-density fluid is well described by the van der Waals (vdW) equation, [*P* + *a*(*N*/*V*)^2^]­[*V*–*Nb*] = *Nk*_B_ *T*, where *a* and *b* are the contributions to the second virial coefficient of the attractive and repulsive part of the pairwise potential, respectively.^55^ Because WCA particles have only repulsion, *a* = 0 and *b*(*T*) = −2π∫_0_^2^1/6^σ^ *dr r* ^2^[*e* ^–*U*_*nb*_(*r*)/(*k*_B_ *T*)^ – 1]. Rearranging the vdW equation, we get *V* = *Nb*(*T*) + (*N*/*P*)*k*_B_ *T*, or

A4
$$L_{z,\mathrm{fluid}}=\mathrm{Nb}\left(T\right)/A+\left[N/\left(\mathrm{PA}\right)\right]k_{B}T$$

Given the steepness of the WCA potential, the temperature dependence of *b*(*T*) is expected to be small; we can approximate it in our temperature range as *b*(*T*) ≈ *b*(0.4ϵ/*k*_B_) ≡ *b*_0_, within an error of less than about 5%. The equation of state then becomes

A5
$$L_{z,\mathrm{fluid}}\left(T\right)\approx Nb_{0}/A+\left[N/\left(\mathrm{PA}\right)\right]k_{B}T$$

which suggests a linear relationship between the length of the box and temperature. For our simulation parameters, we get *Nb*_0_/*A* ≈ 4.21 σ and *N*/(*PA*) ≈ 175σ/ϵ.

Using the Nosé-Hoover (NH) algorithm implemented in LAMMPS, we ran MD simulations of the fluid in the (*N*,*P*_*zz*_ ,*T*) ensemble, using parameters for the thermostat and barostat suggested in the LAMMPS documentation, and obtained *L*_z,fluid_(*T*,*P*_*zz*_ ,*N*, *A*) (Figure S2a). We fit this expression to *L*_z,fluid_ = *aT* + *b* (Figure S2a). The slope *a* is essentially identical to *N*/(*PA*) estimated above, and the intercept *b* is within a few percent of *Nb*_0_/*A* described before. (see Figure S2a and details in the caption). These values were used during the cooling schedule to ensure that at all temperatures the pressure normal to the interface was about *P* = 0.01ϵ/σ^3^.

### A.4 Preparation of a Single Film

To prepare a single layer of polymer melt we follow Auhl et al.,^56^ who used: (i) a short Monte Carlo (MC) simulation to generate the initial conformations of each polymer, (ii) a longer MC simulation in order to homogenize the density within the melt, and (iii) an MD simulation in which the repulsive interactions are slowly “grown” from zero to a full-strength WCA potential, which gently “pushes off” beads that are too close to each other. The major differences in our protocol are as follows.

- First, when enforcing the presence of the interface, polymer conformations with any monomer *i* such that *z*_*i*_ < – *L*_*z*_/2 or *z*_*i*_ > *L*_*z*_/2 were rejected, where *L*_*z*_ is the size of the box in the *z* direction. This creates a polymer film that is periodic in the *x*–*y* plane and surrounded by vacuum in the *z* direction.
- Second, the nonbonded interaction between monomers was slowly added using the soft WCA potential,
  A6
  $$U_{\mathrm{SOFT}}\left(r\right)=\left\{4\epsilon \lambda^{n}\left[\frac{1}{{\left[\alpha {\left(1-\lambda \right)}^{2}+{\left(\frac{r}{\sigma }\right)}^{6}\right]}^{2}}-\frac{1}{\alpha {\left(1-\lambda \right)}^{2}+{\left(\frac{r}{\sigma }\right)}^{6}}\right]+\epsilon \lambda^{n}\right\}\times \Theta \left({\left[2-\alpha {\left(1-\lambda \right)}^{2}\right]}^{1/6}\sigma -r\right)$$
  The potential is null for λ = 0 if *n* > 0, and equals the WCA potential (eq 7) when λ = 1. For a larger *n* the potential grows more suddenly; we used *n* = 1, which is smaller than what is often used when atoms are created or removed.^57^ The smaller *n* provides a more gradual growth of the repulsion. For 0 < λ < 1, the potential is flat, and the repulsive force is near zero when *r* < σ [α­(1 – λ)^2^]^1/6^. This prevents strong repulsion from taking place, but makes the “push off” ineffective for close-by monomers. Thus, we employed a small α = 0.1 σ. These choices are not optimized for performance.
  Following Auhl et al.,^56^ λ was progressively raised from 0 to 1 in either 10^2^ or 10^3^ discrete steps during the simulation. The time between steps should be large enough to allow monomers to be “pushed off”, so that they are not closer than the growing size of the excluded volume core in order to avoid numerical instabilities. At the same time, long simulations (longer than the Rouse time) conducted with small values of λ lead to close contacts that must be removed and, as suggested by Auhl et al.,^56^ it is not clear what the chain statistics would be.
- Third, in order to create an interface, repulsive walls were placed at –(*L*_*z*_ + σ)/2 and (*L*_*z*_ + σ)/2, with functional form given by,
  A7
  $$U_{w\mathrm{all}}\left(z\right)=\left\{\epsilon \left[\frac{2}{15}{\left(\frac{\sigma }{z}\right)}^{9}-{\left(\frac{\sigma }{z}\right)}^{3}\right]+\sqrt{\frac{10}{9}}\epsilon \right\}\Theta \left[{\left(\frac{2}{5}\right)}^{1/6}\sigma -z\right]$$

The MC codes were written *in-house*, while the MD simulations we performed using LAMMPS. Note that we used eq 11 instead of the potential proposed by Auhl et al.^56^ only for ease of implementation within LAMMPS, and not because we believe that it constitutes an improvement over the original approach. At the end of these steps the film comprises KG polymers in a melt confined between walls at density ≈ 0.85 *m*/σ^3^.

To prepare the system, we ran KG polymers at *T* = 1.0ϵ/*k*_B_ for at least 10^5^τ within fixed walls. Next, we modified the interaction between monomers to the one in eq 8 and we run the film for 10^3^τ at fixed temperature using a NH thermostat (using settings suggested in the LAMMPS documentation), and 10^5^τ at constant energy. We cooled down the film to *T* = 0.8ϵ/*k*_B_ by decreasing the temperature over a time 10^3^τ, then for 10^3^τ the temperature was kept at *T* = 0.8ϵ/*k*_B_ using a NH thermostat, and for 10^5^ *τ* at constant energy. This last step was repeated to bring the temperature to *T* = 0.6ϵ/*k*_B_. We then replaced the walls with a fluid pre-equilibrated at *P* = 0.01ϵ/σ^3^ (in order to avoid clashes, we leave a *r*_cut1_/2 padding between the melt and the fluid), and we run for 10^5^τ with NH and 10^5^τ at constant energy. From now on, we refer to the box size in the *z*-direction as *L*_*z*_ = *L*_*z*,melt_ + *L*_*z*,fluid_. Finally, we sampled initial conformations for the double layer system long enough to produce uncorrelated initial samples. In practice, we took conformations separated by Δ*t* = 10^4^τ which is about an order of magnitude larger than the Rouse time *τ*_R_ ≈ 1.3 × 10^3^τ. Here, the symbol ⟨···⟩ refers to an average over all the polymers whose center of mass at time *t* = 0 was within 1 σ from the center of the film.

### A.5 Fixed-Rate Cooling

We cool the system (see Figure 1a) using a NH thermostat, lowering it by Δ*T* = 10^–2^ϵ/*k*_B_ every Δ*t* = 10^4^τ, or a cooling rate of *Γ* = Δ*T*/Δ*t* = 10^–6^ϵ/*k*_B_ × *τ* ^–1^. While the system is cooled down, the pressure of the fluid is kept at approximately *P*_atm_ = 0.01ϵ/σ^3^ by changing the box size in the *z* direction to Δ*L*_film_(*T*) + *L*_*z*_(*T*;*P*_atm_), where *L*_*z*_(*T*; *P*_atm_) is the equation of state of the film described in Section A.3, and Δ*L*_film_ = max_*i*=1,···,*nN*_ *z*_*i*_ – min_*i*=1,···,*nN*_ *z*_*i*_ is the maximum separation between two monomers at extreme ends of the film in the *z* direction. Only the fluid molecules were remapped into the box after this transformation, so that the melt remains undisturbed. We did not find unstable results due to strong clashes between beads, possibly because (i) the density of the fluid is low, and (ii) the typical changes in the box size are small. The dependence of the pressure in the *z* direction on temperature during cooling is shown in Figure S2b, which suggests that the procedure work well, although can be improved.

### A.6 Simulations at Intermediate Temperatures

In order to understand the dependence of polymer dynamics on temperature, after preparing the system at temperatures *k*_B_ *T*/ϵ = 0.6, 0.55, 0.5, and 0.45, we performed constant-energy simulations for 5 × 10^6^τ at the highest temperature and for 10^8^τ at lower temperatures, and we repeated them 5 times. We used these trajectories to compute the autocorrelation function of the end-to-end distance (see Section B.5 [Sec app2-sec16], **Uniform Temperature Simulations**).

### A.7 Construction and Simulation of the Double Layer

To construct the double layer system (see Figure 1b), we follow the idea used in other studies of placing two interfaces as close as possible without particle overlap.^3^

1. We took a conformation of the single layer at temperature *T*_c_ and exposed the top interface (*z* > 0) as follows. We placed a fictitious WCA monomer in a point (*x*,*y*,*z*) with |*x*|< *L*/2, |*y*|< *L*/2 and *z* far above the interface. Then, we moved this along *z* from far above the top interface down to the first point at which the normal force of this monomer was >0. We repeated this calculation for many starting values of *x* and *y* and take the minimum *z* as the deepest trough of the rough interface.
2. We scale all of the coordinates so that the deepest trough is at *z* = 0.
3. After making the necessary changes, we repeated the same procedure to expose the bottom interface of a different conformation at temperature *T*_h_.
4. We combined the two systems by placing them next to each other, which gives two polymer melts surrounded by fluid with strong clashes around *z* = 0 due to the roughness of the interfaces. To relieve these clashes, we used the following procedure. We computed the force *f*_*z*_ between the two melts in the *z* direction, using only the repulsive part (WCA) of their interaction potential. If |*f*_*z*_|> 0, we move the top melt and the top fluid by δ/2, and the bottom melt and fluid by – δ/2. We then repeated the procedure until |*f*_*z*_| = 0. This typically required a total displacement of about *L*_*z*,rough_ ≈ 2 – 4 σ which depends on the specific configurations used for two layers. Note that if the temperatures of the top (*L*_p+1_) and bottom (*L*_p_) layers differ, the top half and the bottom half of the box are of different sizes, *L*_*z*,*p*+1_ = *L*_*z*,*p*+1,melt_ + *L*_*z*,*p*+1,fluid_ and *L*_*z*,*p*_ = *L*_*z*,*p*,melt_ + *L*_*z*,*p*,fluid_, respectively, with *L*_*z*,*p*+1,melt_ ≳ *L*_*z*,*p*,melt_ and *L*_*z*,*p*+1,fluid_ ≳ *L*_*z*,*p*,fluid_, where the ≈ symbol holds when the temperature of the two layers are the same. The total initial box size is *L*_*z*_ = *L*_*z*,*p*+1_ + *L*_*z*,*p*_ + *L*_*z*,rough_. Note that repeating the cooling procedure leads to different *L*_*z*,*p*_ and *L*_*z*,*p*+1_, and that *L*_*z*,rough_ is also conformation-dependent. Hence, *L*_*z*_ varies between different samples.
5. We ran the simulations of the double layer as follows. Because the temperatures of the two layers differ, we could only use Velocity Verlet (no thermostat) to integrate the equations of motion. In order to avoid spurious energy transfer to the fluid, we confined the fluid within repulsive walls (eq 12) placed at *L*_*z*,*p*+1_ + 0.5σ and −*L*_*z,p*_ – 0.5σ, so that the final box size is *L*_*z*_ = *L*_*z*,*p*+1_ + *L*_*z*,*p*_ + *L*_*z*,rough_ + σ, where we have introduced some padding to avoid strong clashes between the fluid and the newly inserted wall.
6. We rescale to *T*_c_ the temperature of the fluid particles whose location in the *z*-direction is given by *z* < 0, and we rescale to *T*_*h*_ the temperature of the fluid particles with *z* > 0. (see Figure 1b). The rescaling is performed every time the temperature of the fluid deviates from the imposed temperature by more than 10^–4^ϵ/*k*_B_. This serves the purpose of providing coupling with a thermostat that can absorbed the energy dissipated by the polymer films. The system is in nonequilibrium and thus its energy drifts until it reaches a stationary value (Figure S7a), and the temperature decreases nearly to *T*_m_ = (*T*_h_ + *T_c_*)/2 = 0.45ϵ/*k*_B_ (Figure S7b). In order to test the effect of thermostatting the fluid, we performed some simulations in which the dynamics of fluid particles was modeled using an energy-conserving (*NVE*, velocity Verlet as implemented in LAMMPS^16^) algorithm. This effectively decouples the system from a thermostat, and the energy is conserved despite the temperature quickly increases and then remains stationary at around 0.47ϵ/*k*_B_ (see Figure S8a and S8b).

Control simulations were performed using top and bottom layers at the same intermediate temperature, *T*_*m*_ = (*T*_*h*_ + *T*_c_)/2, and at a series of other temperatures larger than *T*_*m*_, namely *k*_B_ *T*/ϵ = 0.5, 0.55, and 0.6. We tested simulations in which the fluid is or is not coupled to a thermostat, with the total energy and temperature behaving similar to what was described before (for the system prepared with a thermal gap between the two films); see Figures S7de and S8de.

### A.8 Systems in Vacuum

In a few cases we simulated the polymer films in vacuum, without the surrounding fluid. We either did so during preparation of the films and welding, in the case of thick polymer films (see Appendix A.10 [Sec app1-sec10]), or by removing the surrounding fluid after the juxtaposed pair of films had already been constructed.

### A.9 Removal of Polymer Center of Mass Movement

In the simulations of both the single layer and the double layer, there is momentum exchange between the fluid and the melt, so the center of mass of the melt moves in *x*, *y*, and *z*. In particular, the center of mass in the *z* direction oscillates during the simulations and drifts in the presence of an initial temperature gap between the two films (see Figures S7c,f and S8c,f). We believe that this movement is of no interest in this study, and we have thus removed the center of mass of the polymers from the data analysis.

### A.10 Preparation of the Thick Films

The thick films were prepared by joining together 11 copies of the thin film at *T* = 0.6ϵ/*k*_B_. As a result, the film is made of *N* = 11,000 polymers. The system was modeled in vacuum, and simulations were carried out using a combination of NH, Velocity Verlet, and Langevin dynamics as implemented in LAMMPS (damping coefficient = 1 ^16^). The thick film was allowed to run for almost 10 bulk Rouse times before starting to sample initial configurations for quenching. Quenching was performed using a protocol as described for the thin film, with two differences: (i) we used Langevin dynamics to model the coupling to the environment, (ii) given the size of the system, the quench rate was 10 times faster, *Γ* = 10^–5^ϵ/(*k*_B_ *τ*). Quenching was repeated multiple times, starting from conformations separated by 8000τ, which is nearly 8 Rouse times. The juxtaposition of the films followed a protocol similar to what was done for the thin films. The only difference was that we monitored the overall energy of the system as the two films approach, and we moved them closer in small steps until the energy reached a minimum. Given that the initial values of the OP are the same (see Figure S9d), the two methods are equivalent. Finally, because the density of the glassy system is larger than the density of the melt and the films are thick, the center of mass is far from the interface. Hence, when computing the temperature field of the thick film prepared in the presence of a thermal gradient, instead of removing the center of mass, we rescaled the position of the two films in such a way that the interface was always located at 0σ.

### A.11 Fields

Density ρ­(*r⃗*,*t*) and temperature *T*(*r⃗*, *t*) fields are used to describe the relaxation of the polymer films to equilibrium after they are placed in contact. The statistical mechanical definition of the fields is given in the literature.^58–101^ Here, we take advantage of the symmetry along *x* and *y*, and write the fields only in the *z* direction, or ρ­(*z*,*t*) and *T*(*z*,*t*). Thus, we compute the fields by dividing the polymer films and/or the surrounding fluid in cuboids of area *A* = *L* ^2^ and thickness equal to Δ*z* = 10*σ*, Δ*z* = 2*σ* or Δ*z* = 0.125*σ*, depending on the desired resolution. For the density field we have,

A8
$$\rho \left(z,t\right)=\frac{1}{L^{2}}\left\langle \sum_{\alpha =1}^{N}\sum_{i=1}^{n}m\delta \left[z_{\alpha ,i}\left(t\right)-z\right]\right\rangle \approx \frac{1}{{\left(L\right)}^{*2}\Delta z^{*}}\left\langle \sum_{\alpha =1}^{N}\sum_{i=1}^{n}I_{z,\Delta z}\left[z_{\alpha ,i}\left(t\right)\right]\right\rangle \frac{m}{\sigma^{3}}$$

where the Dirac delta function *δ*[*z*_*α*,*i*_(*t*) – *z*] is approximated as $\delta \left[z_{\alpha ,i}\left(t\right)-z\right]\approx \Delta z^{-1}I_{z,\Delta z}\left[z_{\alpha ,i}\left(t\right)\right]$ , where $I_{z,\Delta z}\left[z_{\alpha ,i}\left(t\right)\right]=1$ for $z_{\alpha ,i}\in \left[z-\frac{\Delta z}{2},z+\frac{\Delta z}{2}\right)$ and 0 otherwise. Similarly, the temperature field is,

A9
$$T\left(z,t\right)=\frac{\frac{1}{3k_{B}L^{2}}\left\langle \sum_{\alpha =1}^{N}\sum_{i=1}^{n}m{\left[{\mathrm{v⃗}}_{\alpha ,i}\left(t\right)-\mathrm{V⃗}\left(z,t\right)\right]}^{2}\delta \left[z_{\alpha ,i}\left(t\right)-z\right]\right\rangle }{\frac{1}{L^{2}}\left\langle \sum_{\alpha =1}^{N}\sum_{i=1}^{n}\delta \left[z_{\alpha ,i}\left(t\right)-z\right]\right\rangle }\approx \frac{1}{3}\frac{\left\langle \sum_{\alpha =1}^{N}\sum_{i=1}^{n}{\left[{\mathrm{v⃗}}_{\alpha ,i}^{*}\left(t\right)-{\mathrm{V⃗}}^{*}\left(z,t\right)\right]}^{2}I_{z,\Delta z}\left[z_{\alpha ,i}\left(t\right)\right]\right\rangle }{\left\langle \sum_{\alpha =1}^{N}\sum_{i=1}^{n}I_{z,\Delta z}\left[z_{\alpha ,i}\left(t\right)\right]\right\rangle }\frac{\epsilon }{k_{B}}$$

where *V⃗*(*z*, *t*) is the streaming velocity field,

A10
$$\mathrm{V⃗}\left(z,t\right)=\frac{L^{-2}\left\langle \sum_{\alpha =1}^{N}\sum_{i=1}^{n}m{\mathrm{v⃗}}_{\alpha ,i}\left(t\right)\delta \left[z_{\alpha ,i}\left(t\right)-z\right]\right\rangle }{L^{-2}\left\langle \sum_{\alpha =1}^{N}\sum_{i=1}^{n}m\delta \left[z_{\alpha ,i}\left(t\right)-z\right]\right\rangle }\approx \frac{\left\langle \sum_{\alpha =1}^{N}\sum_{i=1}^{n}{\mathrm{v⃗}}_{\alpha ,i}^{*}\left(t\right)I_{z,\Delta z}\left[z_{\alpha ,i}\left(t\right)\right]\right\rangle }{\left\langle \sum_{\alpha =1}^{N}\sum_{i=1}^{n}I_{z,\Delta z}\left[z_{\alpha ,i}\left(t\right)\right]\right\rangle }\frac{\sigma }{\tau }$$

Finally, we monitor the space- and time-dependent conformation of the polymers by computing the following field (we will refer to this as “polymer conformation field”),

A11
$$R_{g}^{2}\left(z,t\right)=\frac{L^{-2}\left\langle \sum_{\alpha =1}^{N}R_{g,\alpha }^{2}\delta \left[z_{\alpha }\left(t\right)-z\right]\right\rangle }{L^{-2}\left\langle \sum_{\alpha =1}^{N}\delta \left[z_{\alpha }\left(t\right)-z\right]\right\rangle }\approx \frac{\left\langle \sum_{\alpha =1}^{N}R_{g,\alpha }^{*2}I_{z,\Delta z}\left[z_{\alpha }\left(t\right)-z\right]\right\rangle }{\left\langle \sum_{\alpha =1}^{N}I_{z,\Delta z}\left[z_{\alpha }\left(t\right)-z\right]\right\rangle }\sigma^{2}$$

where *R*_g,*α*_^2^ is the square of the radius of gyration of the *α*-th polymer. Note that *R*_g_^2^(*z*, *t*) in eq 16 can be separated into three components, *R*_g_^2^(*z*, *t*) = *X*_g_^2^(*z*, *t*) + *Y*_g_^2^(*z*, *t*) + *Z*_g_^2^(*z*,*t*), and in the results we focus on *Z*_g_^2^(*z*, *t*)/*R*_g_^2^(*z*, *t*). Errors on the mean are obtained either via error propagation or bootstrapping, resulting in similar estimates.

## B. Polymer Properties

### B.1 Density Dependence on Temperature

To compute the bulk density of the film (ρ_bulk_) as a function of temperature, we repeat the cooling procedure *N*_sample_ times and consider only the conformations just before the next cooling step. Using this ensemble of conformations, we remove the center of mass of the film in the *z* direction and compute ρ­(*z*,*t*) using eq 13. We take the average and standard error on the mean of the resulting density profile and fit it to eq 1. where ρ_bulk_(*T*) is the density in the bulk of the film, *L*_*z*,melt_(*T*) = 2*z̅* is the thickness of the film, and δ­(*T*) is the sharpness of the interface. This equation has only two free parameters because by definition *A*∫_–∞_^∞^ *L*_*z*,melt_ *z*ρ­(*z*) = *mNn*, and it is easy to show that the integral is equal to 2*z̅* ρ_bulk_, and thus ρ_bulk_ *L*_*z*,melt_ *A* = *mnN*, or ρ_bulk_ = *mnN*/(*L*_*z*,melt_ *A*). If we take 2*z̅* *A* = *V* as the volume of the film, and given that *mnN* is a constant, the thermal expansion coefficient *α* = *V* ^–1^ (∂*V*/∂*T*)_*P*_*z*_,*A,nN*_ = −ρ_bulk_^–1^ (∂ρ_bulk_/∂*T*)_*P*_*z*_,*A,nN*_. Here, the pressure *P*_*z*_ refers to the pressure normal to the polymers imposed by the fluid, which we designed to be constant at around *P*_*z*_ = 0.01ϵ/σ^3^ (Figure S2b).

### B.2 Specific Heat

During the cooling phase of the bulk polymer, we record the energy, *E*, and volume, *V* of the system and compute the enthalpy *H̅*(*T*) = *E̅*(*T*) + *P V̅*(*T*) and the heat capacity as *C*_*P*_(*N*, *T*) = ⟨[*H̅*(*T* + *dT*) – *H̅*(*T* – d*T*)]/(2d*T*)⟩, where (·̅) indicates an average over time during cooling (we remove the first 50% of the trajectory at each temperature), and ⟨···⟩ refers to average over multiple repetitions. The standard error on the mean is obtained via bootstrap using the average results obtained for each trajectory. The specific heat is defined as the heat capacity divided by the total number of monomers in the system.

### B.3 Properties at the Interface

We monitor the time-dependent location of the interface and roughness as follows. We create a grid of *N*_*g*_ × *N*_*g*_ (*N*_*g*_ = 100) spheres in the *xy* plane (parallel to the interface) and we “deposit” them on top of the interface by lowering their *z* coordinate until a sphere touches the a monomer *γ* at the surface of the polymer film. This is performed by computing the time at which the sphere gets to a distance 2^1/6^ *σ* from any monomer of the film and selecting the monomer *γ* that is touched first. We refer to these *γ* monomers as “interfacial monomers” (IMs). We then define the location *z̅* of the interface as the average position of the IMs. Care is taken to count each IM only once. The interfacial roughness Δ_RMSD_ is instead computed as the root-mean-squared-deviation (RMSD) of the *z* coordinate of the IMs.

### B.4 Bulk Polymer Melt

We prepare a polymer melt in bulk, with periodic boundary conditions on all sides of the box, using a series of steps similar to those described above for the polymer film. We generate an initial conformation at *T* = 0.6ϵ/*k*_B_ and cool the system at a quenching rate identical to the one used for the polymer film. The only difference is that we use an *NPT* NH (as implemented in LAMMPS, using standard settings) to simulate the coupling to a barostat and a thermostat. The pressure is isotropic, and thus the box is compressed/expanded by the same amount in the three directions. To compare with the polymer film, we compute the pressure of the melt by averaging the energy and isotropic pressure over the second half of the constant-temperature window between two consecutive cooling steps. For the density, we average the density over the same segment of an isothermal section of the quenching between two consecutive cooling steps. The averages of these quantities are obtained by repeating the procedure 10 times starting from the same initial conformation but with different initial velocities and waiting 2 × 10^4^τ before initiating the temperature quench, in order to produce different initial melt structures (the decorrelation time for the end-to-end distance in the bulk at *T* = 0.6ϵ/*k*_B_ is ≈ 1.2 × 10^3^τ). Extended simulations in bulk are carried out at temperatures *k*_B_ *T*/ϵ = 0.6, 0.55, 0.50, and 0.45 for a time = 10^8^ *τ*. Simulations are performed in the *NVT* ensemble using a NH thermostat as implemented in LAMMPS with standard settings and were not repeated. The average pressure during these runs is close to 0.01ϵ/*σ* ^3^, with an error less than 15%.

We performed further simulations in the *NPT* ensemble (same external pressure, *P* = 0.01ϵ/*σ* ^3^) at the same four temperatures, in order to compute the thermal conductivity and diffusivity of the coarse-grained polymer model. Different conformations were generated by sampling 3 (at the 3 highest temperatures) or 7 (at *T* = 0.45ϵ/*k*_B_) independent configurations (separated by more than one Rouse time), and further sampling data for 10^7^ *τ*. We extracted the energy current, using LAMMPS^16^ routines,

A12
$$J_{\alpha }^{e}\left(t\right)=\sum_{i=1}^{\mathrm{nN}}E_{i}v_{i,\alpha }+\sum_{i=1}^{\mathrm{nN}}\sum_{j>i}\left({\mathrm{F⃗}}_{\mathrm{ij}}\cdot {\mathrm{v⃗}}_{j}\right)r_{\mathrm{ij},\alpha }$$

where *E*_*i*_ is the energy of the *i*-th particle, the vectors *v⃗*, *r⃗*, and *F⃗* represent velocity, position, and force of a particle, and the subscript *α* labels Cartesian components. The Green–Kubo expression for the thermal conductivity is,^60^

A13
$$\kappa =\frac{1}{Vk_{B}T^{2}}\int_{0}^{\infty }dt\left\langle J_{\alpha }^{h}\left(t\right)J_{\alpha }^{h}\left(0\right)\right\rangle =\frac{1}{3Vk_{B}T^{2}}\int_{0}^{\infty }dt\left\langle {\mathrm{J⃗}}^{h}\left(t\right)\cdot {\mathrm{J⃗}}^{h}\left(0\right)\right\rangle$$

Here, the heat current *J⃗* ^*h*^ is the energy current in the frame of reference in which the fluid is quiescent,^60^ so we removed the center of mass velocity before any calculation. We sample configurations every 0.01τ, construct the autocorrelation function and integrate it up to 1τ using the trapezoidal rule. The thermal diffusivity is defined as

A14
$$D_{T}=\frac{\kappa }{\rho c_{P}}$$

which we obtain by computing the density and the specific heat from the fluctuations of the enthalpy during a trajectory:

A15
$$c_{P}=\frac{1}{k_{B}T^{2}\mathrm{Nn}}\left\langle \overset{®}{H^{2}\;}\left(T\right)-\mathrm{H̅}{\left(T\right)}^{2}\right\rangle$$

For the system at *T* = 0.45ϵ/*k*_B_, we conducted three longer simulations (total time = 20000*τ* each, averaged indicated by ···) to estimate the specific heat. The results agree with the estimate obtained from the temperature quenching protocol. We obtain errors from the repetitions, which are indicated with the symbol ⟨···⟩. The error was propagated from the specific heat, thermal conductivity, and density in order to obtain the error on the thermal diffusivity.

### B.5 Uniform Temperature Simulations: Polymer Statistics

During longer simulations carried out at uniform temperature for a single polymer film or for the polymer in bulk, we compute the end-to-end vector autocorrelation function,

A16
$$\phi \left(t\right)=\frac{\left\langle {\mathrm{R⃗}}_{\mathrm{ee}}\left(t\right)\cdot {\mathrm{R⃗}}_{\mathrm{ee}}\left(0\right)\right\rangle }{\left\langle {\mathrm{R⃗}}_{\mathrm{ee}}^{2}\right\rangle }$$

where the average is over different polymers and over multiple repetitions of the trajectory.

## Notes

Comparison of the potential used here for nonbonded interactions with other commonly used potentials; details on the algorithm used to impose the normal pressure on the film via surrounding fluid; characterization of thermal transport properties of the polymer model; further characterization of the time-dependent temperature, density, and polymer shape profiles during welding; master curve of surface roughness during welding; details on energy conservation for different algorithms and setups used; dependence of welding on film thickness; temperature dependence of the Rouse time (PDF)

## ∥

Certain commercial equipment, instruments, or materials are identified in this paper in order to specify the experimental procedure adequately. Such identification is not intended to imply recommendation or endorsement by the National Institute of Standards and Technology, nor is it intended to imply that the materials or equipment identified are necessarily the best available for the purpose.

## ¶

Official contribution of the National Institute of Standards and Technology; not subject to copyright in the United States.

## ⊥

To show this, consider that *A*∫_–∞_^∞^ *dz*ρ­(*z*) = *mNn* on the l.h.s. of eq 1. Carrying out the integral on the r.h.s. of eq 1, one obtains 2*z̅* ρ_bulk_ *A* = *mNn*.
