# Supplementary material for: Weld Formation Between Polymer Films Prepared at Different Temperatures: Insights from Molecular Dynamics Simulations
Source: Macromolecules. 2025 Sep 9;58(18):9567–85. doi: 10.1021/acs.macromol.5c00569 (PMC12461930; doi:10.1021/acs.macromol.5c00569)
Supplement: Supplementary file 1 [file ma5c00569_si_001.pdf]

Supporting Information

Weld Formation Between Polymer Films Prepared at Different Temperatures: Insights from Molecular Dynamics Simulations

Mauro L. Mugnai[1,*], Jonathan E. Seppala[2], and Peter D. Olmsted[1,3,*]

[1]Institute for Soft Matter Synthesis and Metrology, Georgetown University, Washington, DC 20057, USA

[2]Materials Science and Engineering Division, National Institute of Standards and Technology, Gaithersburg, MD 20899, USA

[3]Department of Physics, Georgetown University, Washington, DC 20057, USA

[*]Corresponding Authors. MLM: mm4994@georgetown.edu; PDO: pdo7@georgetown.edu

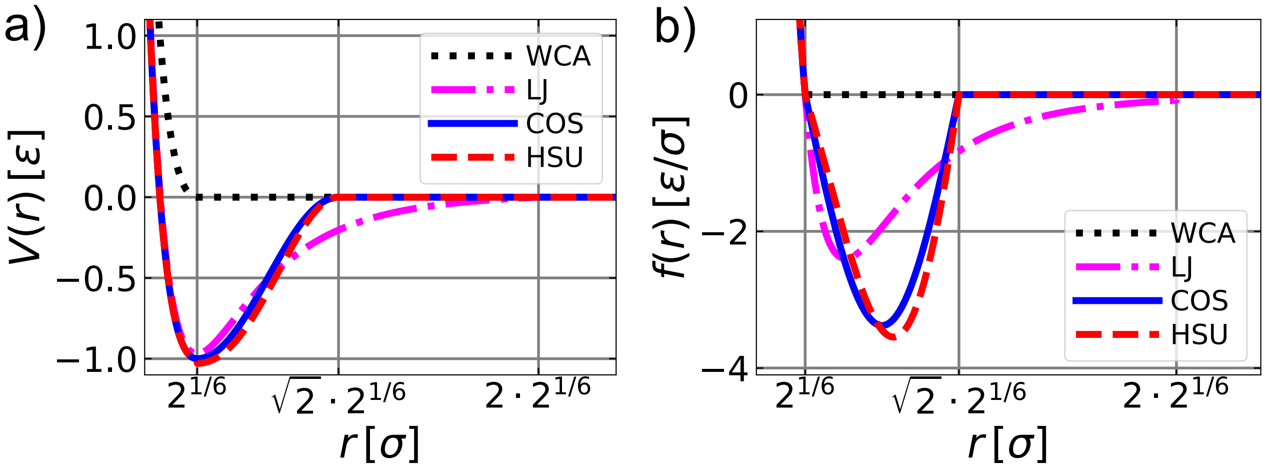

Figure S1: Comparison of different non-bonded energy functions. Panel (a) shows the energy, panel (b) the force, which was obtained as a numerical derivative of panel (a). WCA (black dotted line) is the Weeks-Chandler-Anderson potential, which is $= 0$ after $r_{\text{cut1}} = 2^{1/6}\sigma$. The magenta dash-dotted line shows a Lennard-Jones (LJ) potential truncated at $2 * r_{\text{cut1}}$, as is done in the Bennemann model [1]. The blue continuous line (COS) shows the potential used in this study. The red dashed line (HSU) refers to the Hsu-Kremer potential [2]. Note that as compared with the Hsu-Kremer work, we shifted the repulsive term of the potential to $-\epsilon$ to make it look continuous. This shift has no impact on the force.

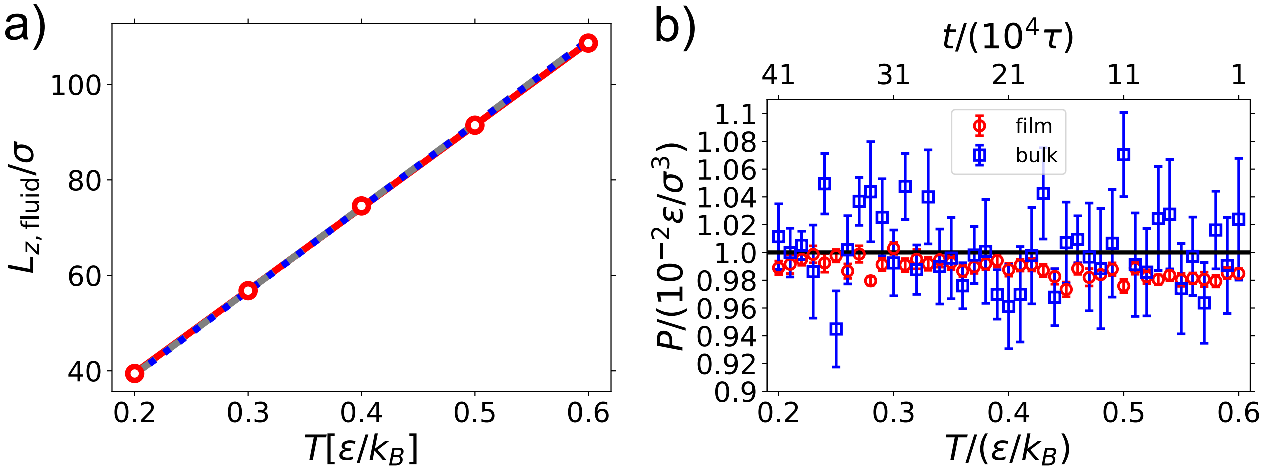

Figure S2: Using the surrounding fluid to control the pressure. (a) Length of a box $L_{\text{z,fluid}}(T)$ of 907 WCA fluid particles with fixed pressure $P_{zz} = 0.01\epsilon/\sigma^3$ and fixed area A in the x-y plane, as a function of temperature. The red line is the optimal linear fit ($L_{\text{z,fluid}}(T) = 173.1T + 4.95$) to the simulations, the gray dashed line is the equation used in simulations (obtained from a previous fit with preliminary data, $L_{\text{z,fluid}}(T) = 174.92T + 4.32$) and the blue dotted line is obtained from the van der Waals equation of state ($L_{\text{z,fluid}}(T) = 175T + 4.21$). The lines are essentially indistinguishable. (b) Red: z component of the pressure of fluid and melt. Blue: isotropic pressure of a polymer melt during quenching. The black line indicates the expected result, $P = 0.01\epsilon/\sigma^3$. Averages and error bars are obtained over multiple repetitions. The top axis indicates the last time before a temperature reduction by $0.01\epsilon/k_B$.

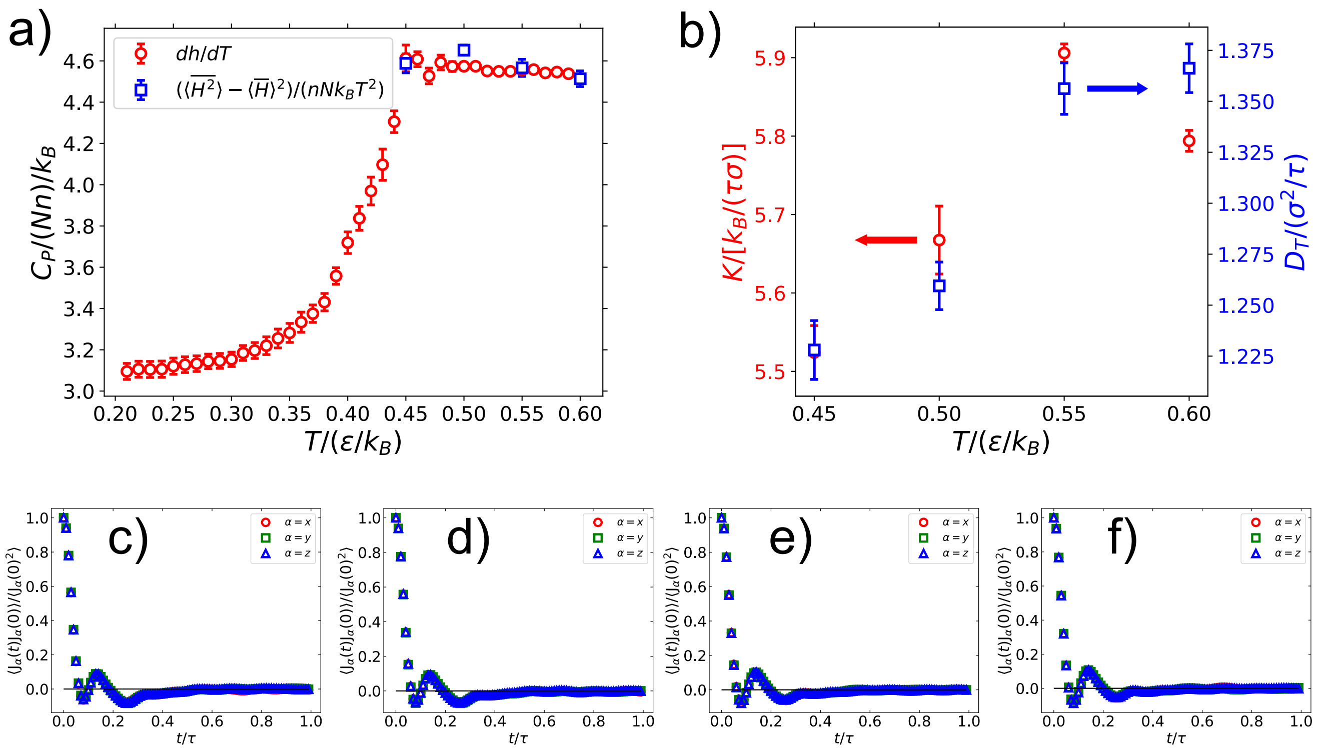

Figure S3: Characterization of thermal transport in the polymer model. a) Specific heat at constant pressure as a function of temperature. Data in red circles were gathered during cooling from $T = 0.6\epsilon/k_B$ to $T = 0.2\epsilon/k_B$ of a bulk system using an isotropic NPT algorithm, with $P = 0.01\epsilon/\sigma^3$. Cooling was performed in steps of $\Delta T = 0.01\epsilon/k_B$ and letting the system relax at a given temperature for $\Delta t = 10^4\tau$, so that the overall quenching rate is $\Gamma \approx \Delta T/\Delta t = 10^{-6}\epsilon/(k_B\tau)$. At every step, the energy and volume of the system were collected during the second half of the relaxation and were average to get the enthalpy $H = \overline{E} + P\overline{V}$, where the overline refers to time average. The data in red circles show the heat capacity obtained as a numerical derivative $C_P(T) \approx [H(T + \Delta T) - H(T - \Delta T)]/(2\Delta T)$, and the specific heat is $c_P(T) = C_P(T)/(nN)$, where n is the length of the polymers and N is their number. Error bars on energy and volume are obtained by repeating the simulations 10 times and are propagated in order to get an error on the specific heat. We performed equilibrium simulations in the NPT ensemble of the system in bulk. Different conformations were generated by integrating the polymer melt for a time larger than the Rouse time at a given temperature. Then, a shorter simulation of $10^4\tau$ was performed to sample the data to compute heat capacity, density, and thermal conductivity (see next). At $T = 0.45\epsilon/k_B$ we repeated the calculation for 3 different initial conditions and for a much longer time $(6 \times 10^5\tau)$ as shorter simulations seemed to underestimate the fluctuations of the enthalpy. For the other temperatures the calculations were repeated 4 times. The blue squares show the specific heat obtained from the fluctuation of the enthalpy $(c_P(T) = (\langle \overline{H^2} - \overline{H}^2 \rangle)/(nNk_BT^2))$ during these simulations averaged over the repetitions by $\langle \ldots \rangle$. b) Thermal conductivity, K (left axis, red dots) and thermal diffusivity, $D_T = K/(c_P\rho)$ (right axis, blue squares) during the above mentioned equilibrium simulations at a subset of temperatures. The thermal conductivity was obtained from the integral of the autocorrelation function of the heat flux computed with LAMMPS [3]. The error bars report the standard error on the mean over the repetitions, which were 7 for $T = 0.45\epsilon/k_B$ and 4 for the other temperatures. Panels (c-f) for show the normalized heat flux autorcorrelation function in three directions (legend) at $T = 0.45\epsilon/k_B$, $T = 0.50\epsilon/k_B$, $T = 0.55\epsilon/k_B$, and $T = 0.60\epsilon/k_B$.

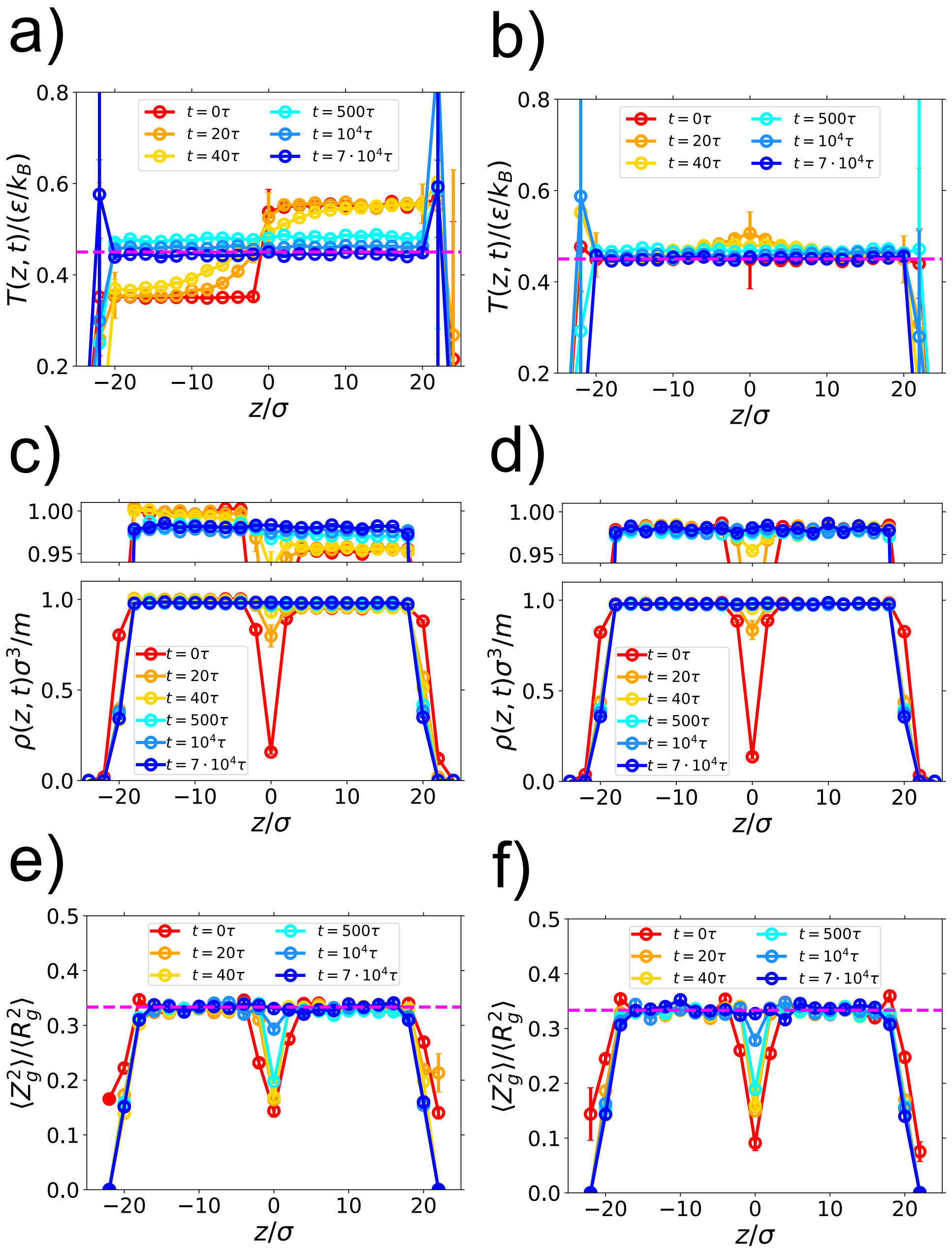

Figure S4: Temperature [(a) and (b)], density [(c) and (d)], and polymer conformation [(e) and (f)] as a function of time t and the coordinate z perpendicular to the interface of the film. Color indicates time, as shown in the legends. The dashed magenta lines in panels (a) and (b) show $T = 0.45\epsilon/k_B$, which is expected after equilibration. The top panels in (c) and (d) enlarge the scale around the bulk value of the density. (e-f) Ratio between the squared component $Z_g^2(z,t)$ of the radius of gyration and the squared radius of gyration for polymers whose center of mass is at z shown in the abscissa. The dashed magenta line shows the value 1/3, expected for bulk polymers. Panels (a), (c), and (e) are obtained from simulations in which L_p and L_{p+1} were prepared at different temperatures while panels (b), (d), and (f) are for control simulations in which L_p and L_{p+1} both begin at $T = 0.45\epsilon/k_B$.

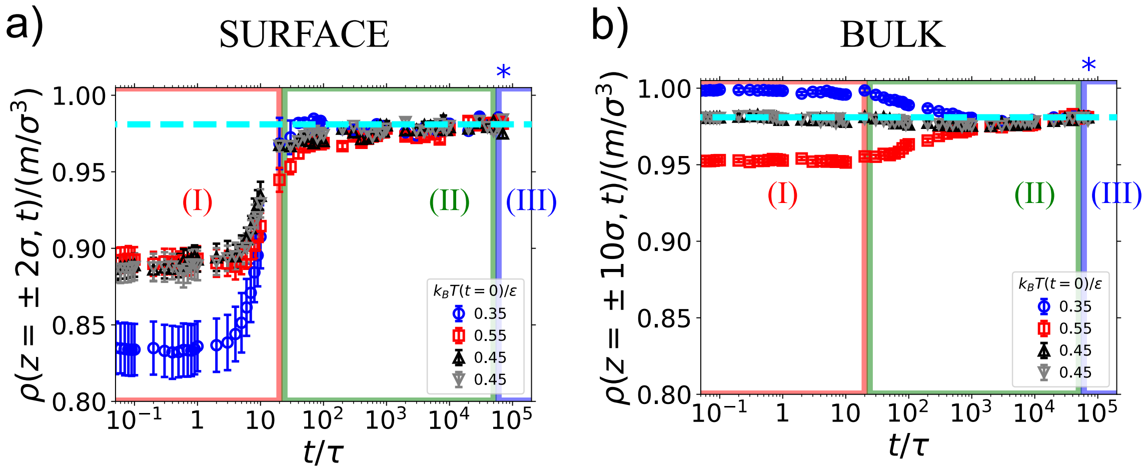

Figure S5: Density at the surface and in bulk as a function of time. (a) Density field computed within 2σ of surface on the hot (red squares) and cold (blue circles) sides, for simulations prepared in the presence of a temperature jump between the two layers. Black and gray triangles are for two layers prepared at the same temperature, and the cyan dashed line shows $0.981m/\sigma^3$. (b) Density in the bulk of the film. For the red squares, the density is averaged over three slabs of thickness $\Delta z = 2\sigma$ and centered at $z = 8\sigma, 10\sigma, 12\sigma$, and around $z = -8\sigma, -10\sigma, -12\sigma$ for the blue circles. Black and gray triangles are for simulations lacking the thermal gradient. The cyan line shows $0.981m/\sigma^3$. In the two panels, the background semi-transparent red, green, and blue boxes identify the three phases of welding: (I) surface approach, (II) surface adjustment, and (III) interdiffusion.

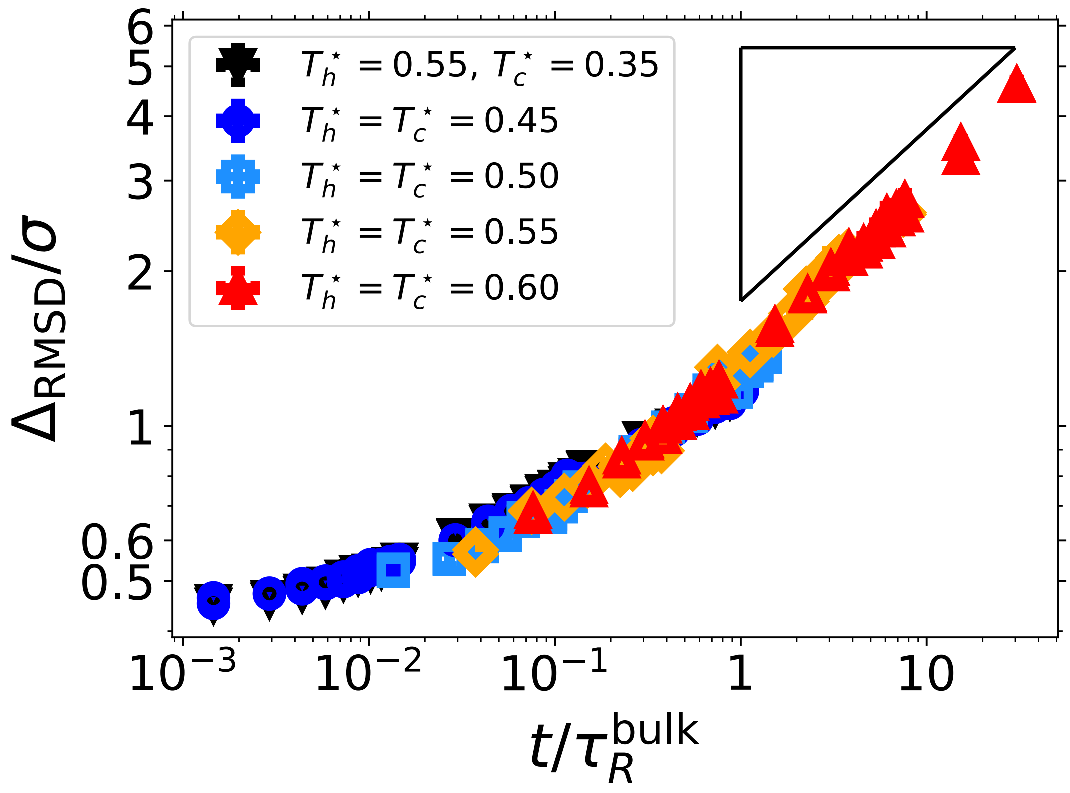

Figure S6: Roughness master curve. Symbols are explained in the legend and indicate different initial setups. The triangle in the top right corner indicates a slope of 1/3.

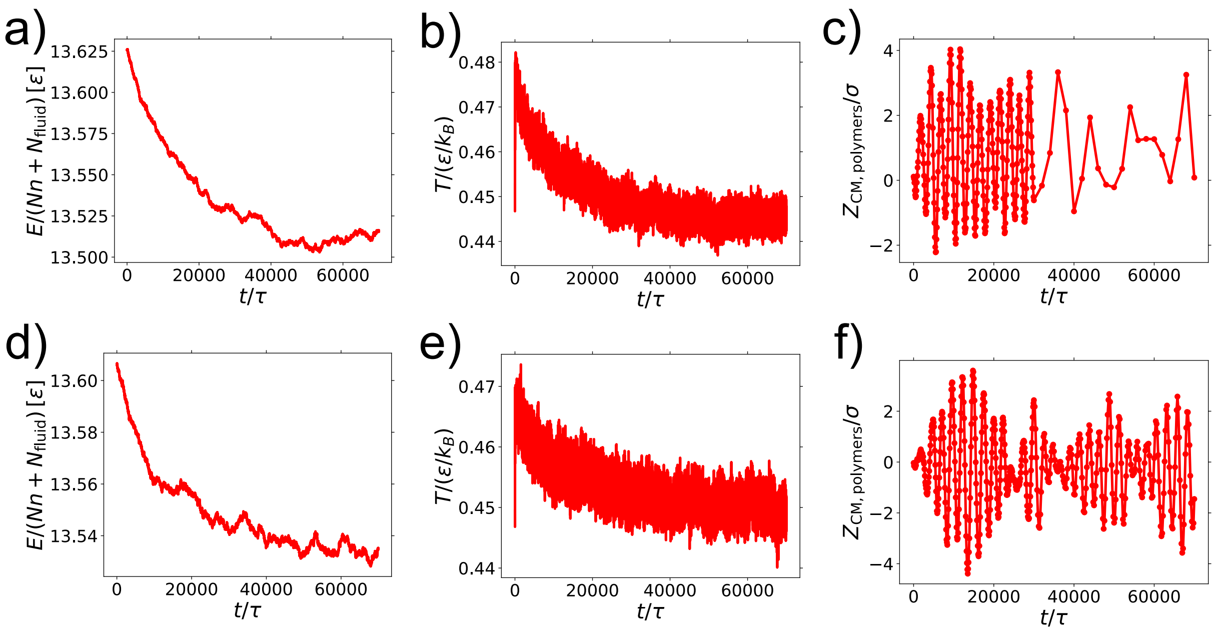

Figure S7: Energy per particle and temperature as a function of time for simulations performed in the presence of fluid coupled with a thermostat. (a-c) refer to a system initiated in the presence of a thermal gradient, that is with top and bottom layers initially set up at $T = 0.55\epsilon/k_B$ and $T = 0.35\epsilon/k_B$. After $t = 3 \times 10^4\tau$ data were saved less frequently. (d-f) are performed at uniform initial temperature, which was set for both layers at $T = 0.45\epsilon/k_B$.

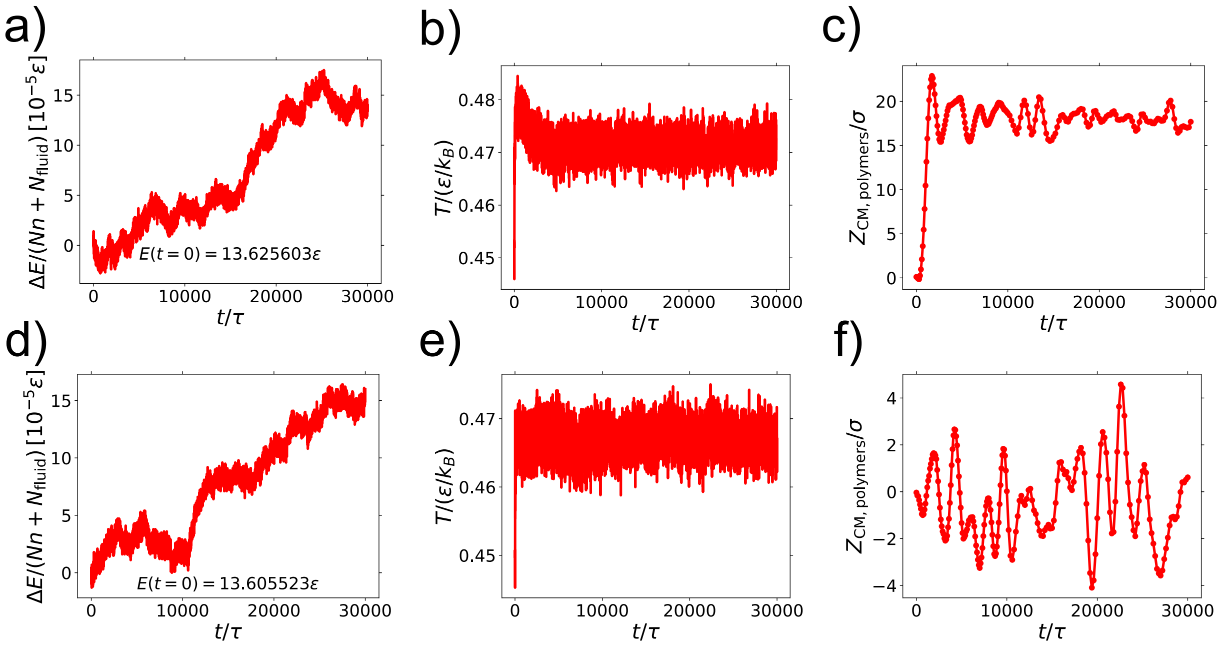

Figure S8: Same as Fig. S7, but for simulations in which the temperature of the fluid was not rescaled to the initial value – that is, energy-conserving simulations. The energies are shown as a deviation from the initial energy in scale of $10^{-5}\epsilon$. The energy at the beginning of the simulation is shown in the figure.

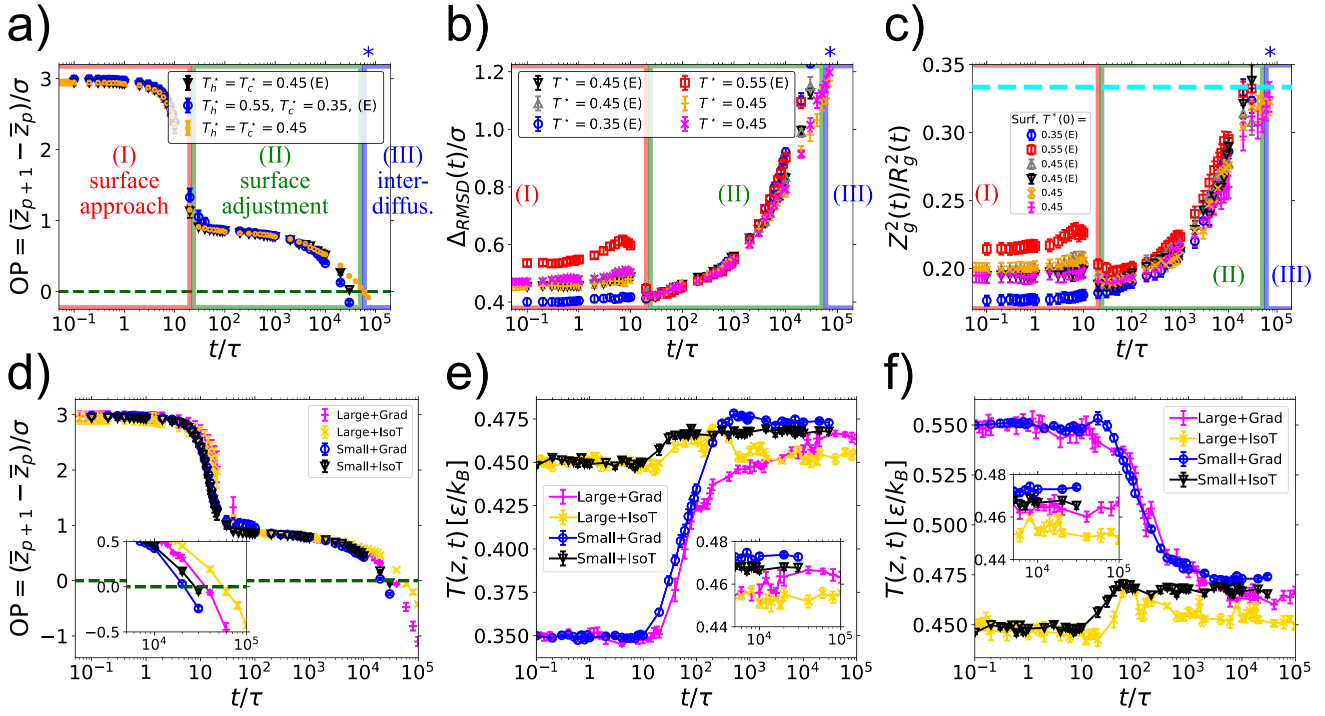

Figure S9: Effect of boundary conditions and system size on welding. (a-c) Comparison of the system with and without thermalization of the fluid. (a) Distance between the two interfaces for the two films prepared with (blue circles) and without (black triangles) thermal gradient. The legend indicates (E) as in energy-conserving. The orange dots refer to simulations in which the fluid was thermalized to its initial temperature. The black triangles here correspond to the orange dots in Fig. 4 of the main text, where we focus only on the initial step. (b) Surface roughness for the two films prepared in the presence of a thermal gradient (blue circle and red square for low and high temperature), and in the absence of a thermal gradient (black and gray triangles). These simulations are labeled (E) because they are energy conserving. The orange plus signs and magenta cross signs refer to isothermal ($T = 0.45\epsilon/k_B$ in both layers) simulations performed with thermalization of the fluid layers. (c) Projection of the radius of gyration in the direction perpendicular to the surface. The color code is the same as in panel (b). The cyan dashed line indicates the value 1/3 attained in bulk. (d-f) Comparison between thin and thick polymer films in vacuum. (d) Order parameter as a function of time for the thick polymer film in the presence of a thermal gradient ($T_h = 0.55\epsilon/k_B$, $T_l = 0.35\epsilon/k_B$, magenta plus signs) and prepared at the same, intermediate temperature ($T = 0.45\epsilon/k_B$, golden crosses), and for the thin film in the presence of the same thermal gradient (blue circles) or prepared at the same intermediate temperature (black triangles). The green, dashed line indicates OP$= 0$, and the inset displays the long-time behavior. (e-f) Temperature profile in a slab of thickness 10σ between -15σ and -5σ (panel e), and between 5σ and 15σ (panel f). The color code is the same as in panel (e). The long-time behavior is displayed in the inset.

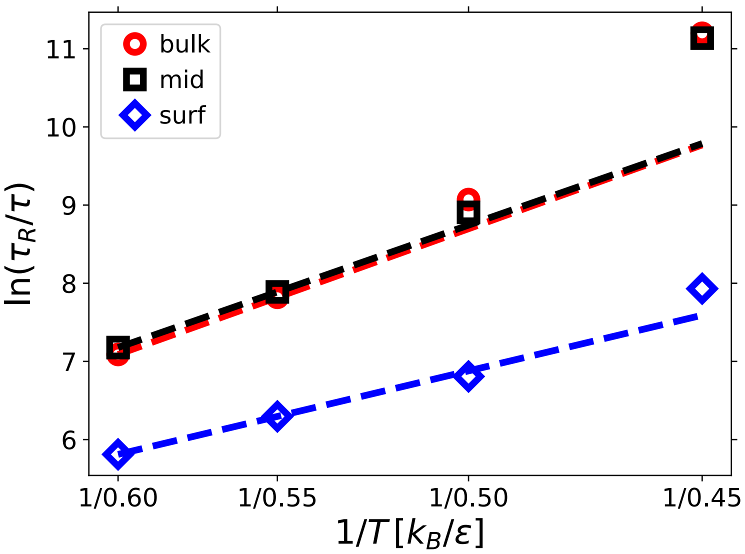

Figure S10: The temperature dependence of the Rouse time for polymers in bulk (red circle), in the middle of the film (black square), and on the surface of the film (blue diamond). The figure shows the logarithm of the Rouse time plotted as a function of the inverse temperature. The dashed lines represent the linear behavior obtained from the two points at the highest temperature. The linear behavior would be expected for a Arrhenius-like dependence of the Rouse time, ie $\tau_R \sim e^{A/T}$. Clearly, at least for polymers in bulk and in the middle of the film, this is not the case, and the curve is better described by $\tau_R \sim e^{A/(T-T_0)}$, which is the so-called Vogel-Fulcher-Tamman (VFT) curve, where $T_0 > 0$ represents the temperature at which $\tau_R \to \infty$.

References

1. Bennemann C, Paul W, Binder K, and Dünweg B. Molecular-Dynamics Simulations of the Thermal Glass Transition in Polymer Melts: α-Relaxation Behavior. Phys. Rev. E 1 1998;57:843–51.
2. Hsu HP and Kremer K. Glass transition temperature of (ultra-)thin polymer films. J. Chem. Phys. 2023;159:071104.
3. Thompson AP, Aktulga HM, Berger R, et al. LAMMPS - a flexible simulation tool for particle-based materials modeling at the atomic, meso, and continuum scales. Comput. Phys. Commun. 2022;271:108171.
